# A hybrid unsupervised methodology on artificial intelligence filtering for automatically processing cellular DNA-encoded library (DEL) datasets

**DOI:** 10.1093/bioinformatics/btag001

**Published:** 2026-01-07

**Authors:** Yiran Huang, Xiao Tan, Xiaoyu Li, Feng Xiong, Siu Ming Yiu

**Affiliations:** School of Pharmacy, Shenzhen University Medical School, Shenzhen University, Shenzhen 518060, China; Department of Computer Science, The University of Hong Kong, Hong Kong SAR, 999077, China; Department of Chemistry and State Key Laboratory of Synthetic Chemistry, The University of Hong Kong, Hong Kong SAR, 999077, China; Laboratory for Synthetic Chemistry and Chemical Biology Limited, Health@InnoHK, Innovation and Technology Commission, Hong Kong SAR, 999077, China; Department of Chemistry and State Key Laboratory of Synthetic Chemistry, The University of Hong Kong, Hong Kong SAR, 999077, China; Shenzhen NewDEL Biotech Co., Ltd., Shenzhen, 518110, China; Department of Computer Science, The University of Hong Kong, Hong Kong SAR, 999077, China

## Abstract

**Motivation:**

DNA-encoded library (DEL) technology has been developed as a powerful platform for drug development. Live cell-based selection methodologies were recently developed to expedite drug candidate discovery with higher biological relevance. Nevertheless, hit characterization is challenged by prominent background signals of cell-based selections. Therefore, automated data processing streamline compatible with noisy sequencing output is highly desirable.

**Results:**

Herein, we report an innovative automatic method that enables the most promising hit identification from large quantities of cell-based DEL datasets with improved accuracy and efficiency. This processing workflow is based on a comprehensive unsupervised algorithm incorporating data pre-processing, feature extracting and outlier filtering, descriptor-based classification, similarity score ranking, and active compound prediction. We performed methodology development with two DEL selection datasets targeting insulin receptor (INSR) on live cells, from both ∼30 million- and 1.033 billion-membered libraries. The automated scheme has demonstrated high consistency with experimental results as well as self-adaptivity to on-cell DEL datasets with varied library scales. Extended methodology application to cellular thrombopoietin receptor (TPOR) further substantiated the algorithmic generalization capability regarding target proteins. Thus, this approach can serve as a widely applicable workflow automatically differentiating hit compounds and thereby facilitates drug development from candidate discovery.

**Availability and implementation:**

The complete datasets, source code, and pre-trained models are made available at https://doi.org/10.5281/zenodo.17452392 and https://doi.org/10.5281/zenodo.17569557.

## 1 Introduction

**Figure 1:**
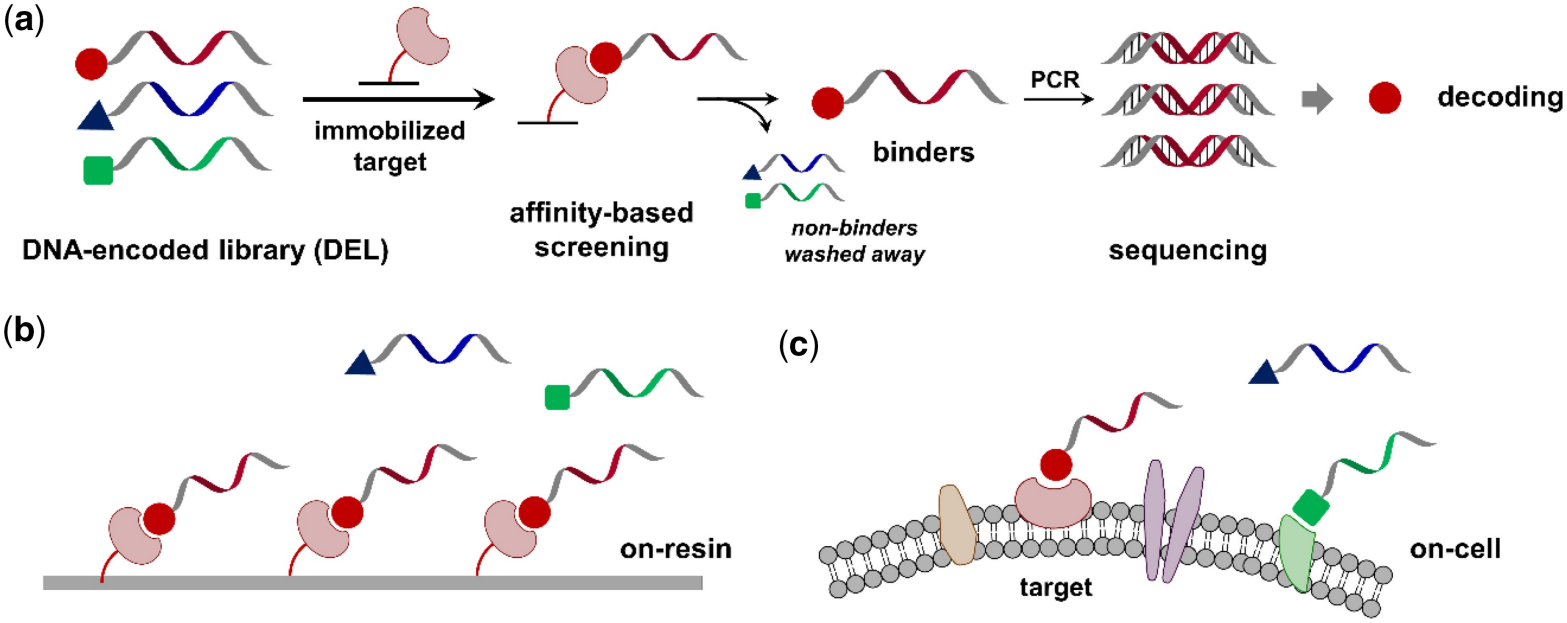
(a) Scheme of typical DEL selections against purified and immobilized proteins. (b) DEL selections with on-resin proteins. (c) DEL selections with cell surface proteins.

DNA-encoded chemical libraries (DELs or DECLs) have become a powerful tool in drug discovery, particularly for early hit identification. In DELs, each library member is conjugated to a unique DNA identifier to encode their chemical structures. Their ability to screen vast numbers of compounds simultaneously on a miniature scale makes them highly attractive for high-throughput screening (Brenner and Lerner 1992, Needels *et al.* 1993, Salamon *et al.* 2016, Goodnow and Davie 2017, Yuen and Franzini 2017, Kunig *et al.* 2018, Neri and Lerner 2018, Kodadek *et al.* 2019, Reddavide *et al.* 2019, Flood *et al.* 2020, Madsen *et al.* 2020, Song and Hwang 2020, Conole et al. 2021, Fitzgerald and Paegel 2021, Gironda-Martínez *et al.* 2021, Kunig *et al.* 2021, Satz *et al.* 2021, Huang *et al.* 2022, Plais and Scheuermann 2022, Satz *et al.* 2022, Sunkari *et al.* 2022, Dixit *et al.* 2023, Dockerill and Winssinger 2023, Matsuo *et al.* 2023, Peterson and Liu 2023). Typically, DEL selections are performed against purified proteins on a solid matrix, with selection outcome based on binding affinities (Fig. 1a). Given that the structures and functions of immobilized proteins may be deviated after the biochemical treatments, performing selections directly under cellular contexts is highly desirable. In recent years, the target scope of DEL selections has been expanded to more biological relevant entities, such as proteins in cell lysates (Harris and Winssinger 2005, Winssinger and Harris 2005, McGregor *et al.* 2010, 2014, Zhao *et al.* 2014, Denton and Krusemark 2016, Chan *et al.* 2017, Shi *et al.* 2017, 2019), membrane proteins on cell surfaces (Wu *et al.* 2015, Cai *et al.* 2019, Huang *et al.* 2021, Oehler *et al.* 2021, Cai *et al.* 2023, Huang *et al.* 2024), intracellular proteins within live cells (Cai *et al.* 2019, Petersen *et al.* 2021), whole bacteria (Yan *et al.* 2019, Cochrane *et al.* 2021), and antibodies in human sera (Mendes *et al.* 2017). These cell-based approaches can significantly enhance the biological potential of drug discovery efforts targeting membrane proteins.

Cell-based DEL selections are often associated with high noise levels, low binder enrichment, and limited reproducibility due to the complex landscape of cellular circumstances (Fig. 1c). To reduce false positives, researchers have employed strategies such as target overexpression (Wu *et al.* 2015, Cai *et al.* 2019, Oehler *et al.* 2021, Petersen *et al.* 2021), DNA “beacons” (Huang *et al.* 2021), or affinity tags (Huang *et al.* 2021). However, it is still challenging to exclude nonspecific binders and identify specific ligands in cell-based selections compared to protein-based selections (Fig. 1b and c) in post-selection data processing. Compounds showing selective high enrichments are generally considered as promising hits, while nonspecific binders are manually classified after selection (McCloskey *et al.* 2020, Torng *et al.* 2023). In this aspect, manual data processing may introduce false positives or false negatives (McCloskey *et al.* 2020). Additionally, various data processing methods have been developed to address the challenges posed by noisy selection datasets. These methods leverage statistical tools (Satz 2015, 2016, Satz *et al.* 2017, Amigo *et al.* 2018, Denton *et al.* 2018, Kuai *et al.* 2018, Faver *et al.* 2019, Gerry *et al.* 2019, Foley *et al.* 2021, Rama-Garda *et al.* 2021, Zhu *et al.* 2022) and artificial intelligence (AI)/machine learning (ML) techniques (Komar and Kalinic 2020, McCloskey *et al.* 2020, Binder *et al.* 2022, Lim *et al.* 2022, Ahmad *et al.* 2023, Chen *et al.* 2024, Hou *et al.* 2023, Torng *et al.* 2023, Zhang *et al.* 2023) for improving both efficiency and accuracy in identifying bioactive compounds, whereas their implementation to cell-based selections has yet to be verified. Recently, our group performed a proof-of-principle study in which an ML-based approach was developed to process cell selection datasets (Hou *et al.* 2023). This approach uses a Maximum A Posteriori estimation loss function and was later adapted to facilitate hit identification from large-scale DEL selections on live cells (Huang *et al.* 2024).

Nevertheless, most existing methods require to tune the program parameters manually. The lack of automation limited their applications to DEL selection datasets against different targeted proteins. Herein, we report a novel unsupervised methodology on AI filtering that mimicked filtering logic in manual data processing but could be performed automatically with enhanced accuracy and efficiency. This method enables characterization of potential active compounds from unlabeled DELs dominated by inactive entries. We have established this approach using a 30.42-million-membered DEL dataset and further demonstrated it with a larger dataset comprising 1.033 billion compounds against the cell surface protein insulin receptor (INSR). The prediction outputs were corroborated by the experimental results showing that these compounds were able to activate INSR on live cells (Huang *et al.* 2024). We have also applied it to process a 30.42-million dataset for another cell-supported target thrombopoietin receptor (TPOR), and valid TPOR agonists were successfully obtained from the outcome.

## 2 Methods

This method adopts the unsupervised ML techniques to accommodate large unlabeled DEL data. ML is generally categorized into supervised and unsupervised learning. Supervised methods, such as random forest (RF) (Ballester and Mitchell 2010, Kinnings *et al.* 2011, Imani and Pardamean 2024), support vector machines (SVMs) (Guo *et al.* 2008, Matsumoto *et al.* 2016, Sengupta *et al.* 2024), and Naïve Bayes classification (Li *et al.* 2019), rely on labeled training data (features *x* and labels *y*) to predict protein–ligand interactions. Unsupervised techniques, such as *k*-means clustering, excel in clustering unlabeled data, enabling automatic ADME/pharmacokinetic prediction (Belkadi *et al.* 2021), drug repurpose (Cong *et al.* 2022), and optimized drug discovery (Hadipour *et al.* 2022). Despite their successful applications in drug development, supervised algorithms struggle with imbalanced datasets, while unsupervised methods like *k*-means clustering lack robust denoising capabilities, limiting their utility in DEL selection datasets.

**Figure 2:**
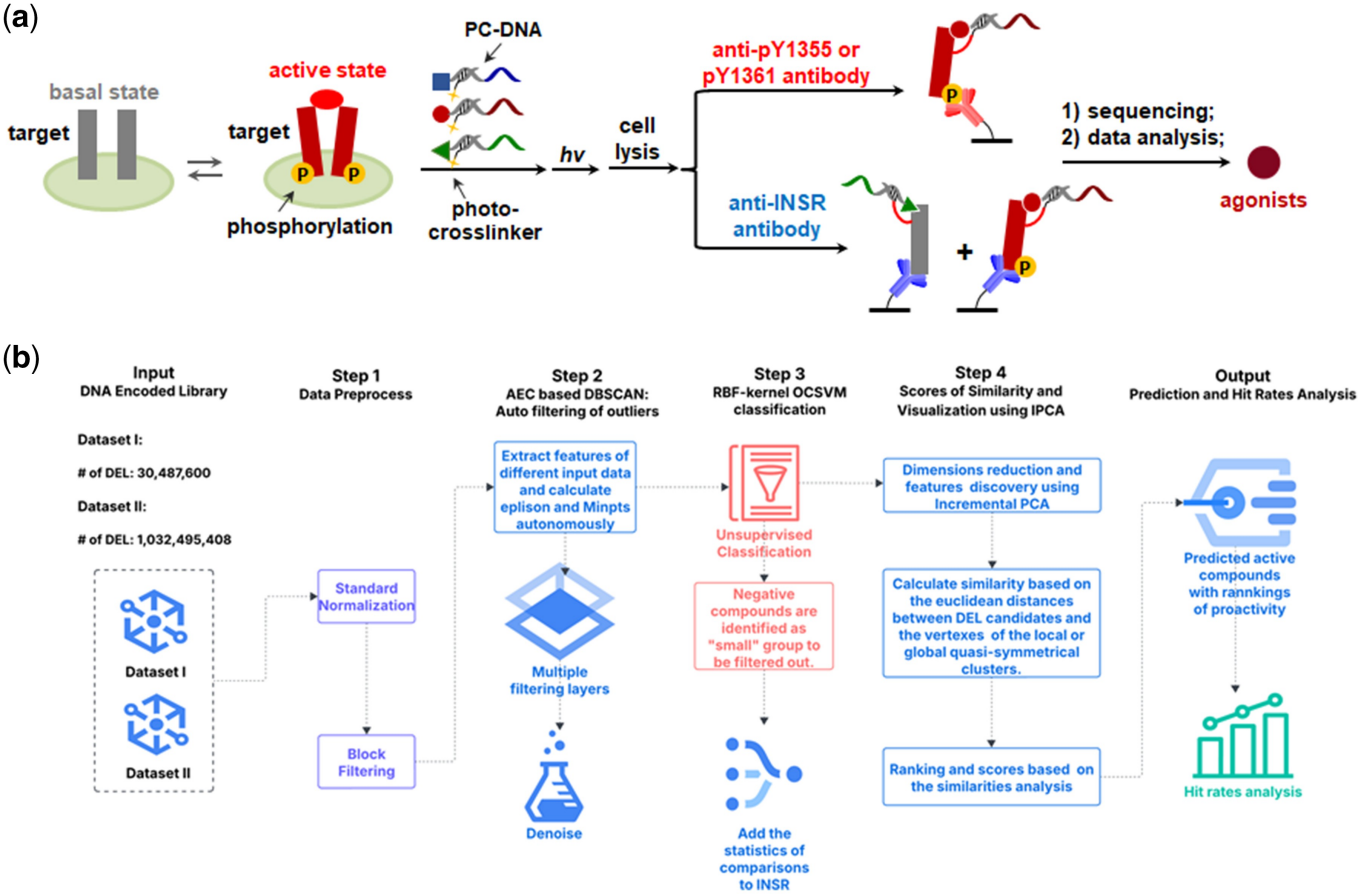
Architecture of the DEL-AI framework. (a) Scheme of the cell-based selections against INSR. (b) Workflow of the hybrid AI framework for DEL data processing.

Therefore, we advanced a hybrid AI framework combining unsupervised classification with automated outlier filtering to identify and prioritize the most promising hit candidates. As illustrated in Fig. 2b, the workflow contains the following steps: (i) **data pre-processing**, where standard normalization and blocking-based filtering are adopted for post-selection DEL dataset; (ii) **auto-filtering of outliers**, where candidates that outperform the average across the entire dataset are fed into the Density-Based Spatial Clustering of Applications with Noise (DBSCAN) algorithm to eliminate outliers derived from diverse selection performance descriptors; (iii) **compound classification**, where the DEL dataset is classified into inactive and potential-active categories using the One-Class Support Vector Machine (OCSVM), an unsupervised learning algorithm designed for scenarios with unlabeled data; and (iv) **similarity scoring and visualization**, where similarity scores are computed based on Euclidean distances between DEL compounds and both local cluster centers as well as global vertices identified through Incremental Principal Component Analysis (IPCA) projection. The similarity metrics eventually form the basis for calculating rankings and activity scores for each potential-active DEL candidate, allowing systematic prioritization of the most promising compounds. The effectiveness of this multi-stage analytical framework is evaluated through comprehensive performance assessment and hit-rate analysis across validation cohorts.

## 3 Results

### 3.1 Dataset information and selection performance descriptors

In a previous study, we selected a 30.42 million- and a 1.033 billion-membered tripeptide DEL, respectively, against INSR overexpressing HEK293 cells using a photo-crosslinking strategy (Fig. 2a) (Huang *et al.* 2024). The photo-crosslinking established covalent bonding between the proteins and their ligands, thus capturing the binding events and significantly enhancing the signal-to-noise ratio experimentally (Denton and Krusemark 2016, Cai *et al.* 2019). Following cell lysis, we employed two distinct antibody-based isolation strategies: (i) anti-INSR antibodies to extract all potential binders, and (ii) phospho-specific antibodies (anti-INSR pY1355 or anti-INSR pY1361) to selectively separate ligands that preferentially bind to the active conformation of INSR, thereby enriching potential agonists. Each DEL selection was performed in 5–6 independent biological replicates. The recovered samples were subjected to PCR amplification and subsequently next-generation sequencing, and the resulting sequencing data were collected as the primary input in the following data processing. We first adopted the 30.42 million-membered DEL selection outputs to facilitate our DEL-AI workflow construction and optimization, and then applied the 1.033 billion-derived dataset to validate the generality of our method across different datasets.

The abundance counts (C), presented by the DNA sequence counts in the post-selection DEL, was defined as the Tier 0 Descriptor to recapitulate the recovery features of library individuals. The compound richness (R) was calculated by (post-selection%)/(pre-selection%) and defined as the Tier 1 Descriptor as it was obtained directly from abundance count. To conduct in-depth analysis for binding patterns of the library compounds, we established a modified statistical descriptor system named Selection Performance Descriptors to extract the target-interacting information for each library member across different replicates (Table 1). Derived from chemical descriptors, the Selection Performance Descriptor model covered all metrics required, such as sum, standard deviation (STDEV) and the average over STDEV.

**Table 1:** Selection performance descriptors for each DEL member.

Symbol	Selection performance descriptors
R	Richness
C	Abundance count
$S_{R}$	Sum of richness
$S_{C}$	Sum of abundance count
$S_{nR}$	Sum of richness reproducibility
$S_{nC}$	Sum of abundance count reproducibility
$\sigma_{R}$	Standard deviation of richness for a DEL compound
$\sigma_{C}$	Standard deviation of the abundance count for a DEL compound
$\Phi_{\mathrm{eff}}$	Average richness over abundance count among repeats
${Κ}_{R}$	Average richness over standard deviation among repeats
${Κ}_{C}$	Average abundance count over standard deviation among repeats
$\Phi_{\mathrm{bal}}$	Absolute value of logarithm of the richness stability to the abundance count stability

With our Selection Performance Descriptor system, we extracted Tier 2 and Tier 3 descriptors based on Tier 0 and Tier 1 Descriptors, to characterize their selection performance in different dimensions, and such descriptors were input for the subsequent normalization, filtering, and clustering algorithms (Toropova and Toropov 2023). Here, we introduced six Tier 2 descriptors and four Tier 3 descriptors to indicate the effectiveness, reproducibility, and stability properties of the DEL compounds against the targeted protein. The involved Tier 2 and Tier 3 descriptors are listed in Table 1, available as supplementary data at *Bioinformatics* online. For validity purposes, we included only DEL compounds with non-zero values for the sum of richness, abundance count, and standard deviation when calculating their Tier 3 descriptor properties.

### 3.2 Data pre-processing

Given that the post-selection DEL datasets, on account of the highly complex cellular landscape, yielded in highly deviated abundance counts and richness across biological replicates, the descriptors calculated from the naïve data could also vary widely in value ranges. As attributes with smaller magnitudes may be overshadowed by those with larger values, these descriptors can compromise model performance in the subsequent data processing steps, especially for OCSVM classification. To address this issue, we implemented standard normalization to the descriptors prior to clustering analysis and scaled the original data to a consistent range between −1 and 1. That is, the standardization was applied before DBSCAN, IPCA, and OCSVM (de Souto *et al.* 2008). This normalization step ensures that each feature contributes equally to the distance calculations used in the OCSVM algorithm, preventing attributes with smaller numerical values from being effectively marginalized during the classification process. Consistent feature scaling is critical for optimizing the performance of these methods across hierarchical algorithmic implementations.

Subsequently, we applied a filtering algorithm onto the normalized descriptors. Albeit proceeded with normalization, the selected DEL datasets still exhibited statistical instability from excessive data size, high value heterogeneity, and sequencing errors. These characteristics can reduce the reliability of classification models in extracting potentially active molecules. In DELs, the compounds was constructed by the assembly of building blocks, and structures with similar building blocks often share comparable binding properties (Zhang *et al.* 2023). Accordingly, we adopted an analogous approach to group descriptors into blocks, termed Block of Descriptors Filtering (BDF). This method introduces the concept of binding blocks and leverages the coupling relationships between abundance count- and richness-related descriptors in DEL datasets.

The original binding blocks Tier 0 and Tier 1 descriptors (i.e. abundance count and richness, respectively) were not directly applied to the DEL filtering; instead, Tier 2 descriptors $S_{R}$, $S_{C}$, $S_{nR}$ and $S_{nc}$, and Tier 3 descriptors ${Κ}_{R}$ and ${Κ}_{c}$ were included as the filtering criteria in extracting statistically significant candidates. We reason that, if a library member only presents binding affinity in one selection but not in other replicates, they are not likely to be a positive candidate; thus, the abundance counts and the richness could not represent the binding performance on their own. In our method, $S_{R}$ and $S_{c}$ were adopted to indicate the effectiveness, $S_{nR}$ and $S_{nc}$ to indicate the reproducibility, and ${Κ}_{R}$ and ${Κ}_{c}$ to indicate the balance of the studied molecule. Accordingly, even high richness or abundance count was occasionally observed in one selection replicate, results from other replicates will dilute its influence in $S_{R}$ and $S_{c}$, while ${Κ}_{R}$, ${Κ}_{c}$ will be further lowered after balanced with large STDEV. The binding block-based filtering was carried out both at level 0 and level 1 algorithm to extract high-confidence data. At level 0 algorithm, all the six descriptors were visited for each library members, while $\sigma_{R}$ and $\sigma_{c}$ were introduced and emphasized at level 1 algorithm so that the compounds that could not stably bind to the target were filtered out. As a result, the BDF rules in level 1 algorithm further enhanced filtering efficacy by focusing on the most reproducible compounds.

### 3.3 AEC-based DBSCAN filtering automatically excluded outliers according to designated descriptors

After applying the BDF to the DEL dataset, potentially active candidates with high-confidence were retained in the filtered dataset and advanced to the following processing stage. While BDF has effectively removed most background signals, residues might remain and potentially influence the location of the SVM hyperplane. This distortion can compromise the accuracy of score computations and the rankings for active components. In response, we integrate DBSCAN to further denoise the filtered dataset. DBSCAN is an unsupervised clustering algorithm independent from pre-labels of the training data, and only minimal hyperparameters need to be tuned in this algorithm. Therefore, DBSCAN is particularly advantageous to anomaly detection for unlabeled datasets with multiple features sets (Parimala *et al.* 2011). As variations in input features were often observed in cell-based DEL selections across different replicates, the common hard-coded architecture of traditional clustering algorithms might fail to achieve consistent predictive accuracy. To address this issue, to the DBSCAN we innovatively introduced the Automatic *Eps* Calculation (AEC) method, where the parameterized architecture will be recalculated whenever it was fed with new data input, thus enabling robust and self-adaptive outlier detection specific to each dataset (Tan and Yiu 2024). On the other hand, the simplicity in DBSCAN parameter requirements also facilitates the automated hyperparameter optimization (HPO) critical for iterative denoising across diverse feature sets (e.g. richness and abundance count, STDEV, and balance indicators).

For proof of principle, the AEC-based DBSCAN was implemented to the filtered dataset from the 30.42-million-membered DEL and automatic outlier filtering were conducted from three distinct layers: the efficiency descriptor $\Phi_{\mathrm{eff}}$, the balance descriptor $\Phi_{\mathrm{bal}}$, and an array of Tier 3 descriptors [${Κ}_{R}$,${Κ}_{C}$] for the five DEL selection replicates. As most deviated data points have been excluded during BDF, only two outliers, **132-172-139** and **56-4-192**, were identified and removed in the DBSCAN step. To estimate the filtering performance of AEC-based DBSCAN, we traced back the molecular behavior of the two outliers during selection. Compound **56-4-192** was located near the lowest point of the anti-pY1355 to anti-INSR richness, seemingly under a binding pattern similar to the neighboring molecules. However, self-adaptive DBSCAN has detected an abnormally large [${Κ}_{R}$,${Κ}_{C}$] for **56-4-192** and thus defined it as an outlier. For **132-172-139**, it deviated from the majority in the original post-selection dataset and was labeled as outlier by DBSCAN according to its efficiency indicator (Fig. 2, available as supplementary data at *Bioinformatics* online). The overall clustering results could also be improved after outlier removal afterwards. In practice, all dimensional descriptors can be utilized either individually or as a combined feature array for filtering, ensuring that outliers with significant impacts on predictive results are effectively excluded. These filtering layers enhance the quality of input data by removing background noise, thereby improving the accuracy of subsequent labeled classifications or cluster assignments. Through practical experimentation, we found that normalizing Tier 3 descriptors into an array significantly improves auto-filtering robustness, particularly when noise has a pronounced impact on predictive performance.

### 3.4 RBF kernel-based OCSVM classification detected the possibly active compounds

**Figure 3:**
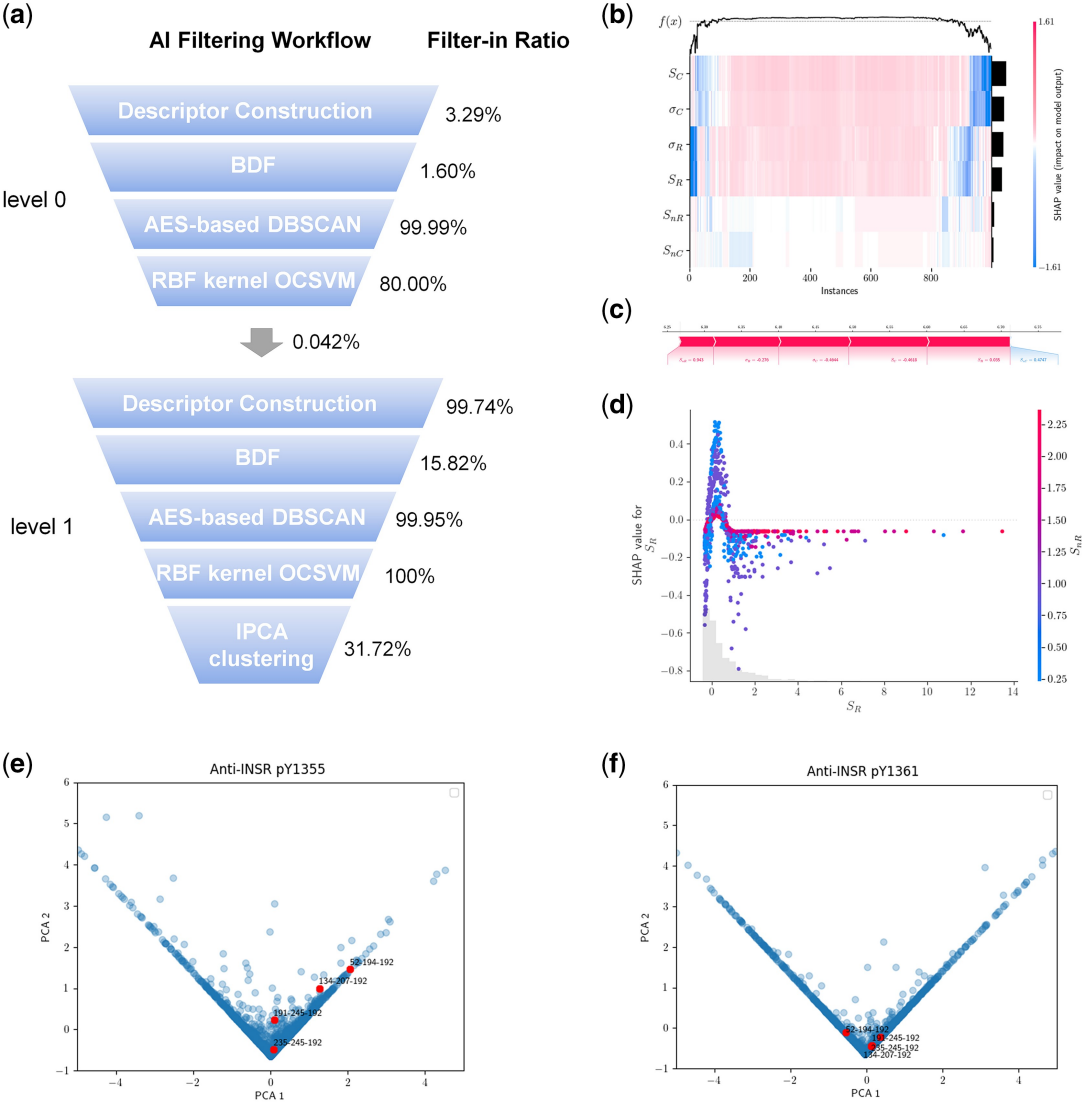
Dual levels of AI filtering enhance the prediction for the most proactive compounds in the 30.42 million-membered DEL on INSR. (a) Stepwise AI filtering results in level 0 and level 1 algorithms. More detailed flowchart with exact compound remaining at each step is shown in Fig. S1, available as supplementary data at *Bioinformatics* online. (b) Heatmap analysis ordering by absolute mean of SHAP values. (c) Force plot analysis of a sample coalition. The *x*-axis represents final predicted SHAP values, with contribution of individual features shown as a block. (d) Dependencies of SHAP values of $S_{R}$. (e) IPCA clustering based on anti-pY1355 data against anti-INSR data before similarity analysis. (f) IPCA clustering based on anti-pY1361 data against anti-INSR data before similarity analysis. *x*-axis recapitulates compound features in anti-INSR selection; *y*-axis recapitulates compound features in anti-phosphorylated INSR selection. Example compounds were highlighted in red.

Next, molecular classification was carried out to differentiate positive compounds. The radial basis function (RBF) kernel-based OCSVM was adopted as the classifier in the method due to its simplicity, effectiveness, and interpretability as an unsupervised machine learning algorithm for classifying nonlinear and high-dimensional features. OCSVM was initially proposed by Schölkopf *et al.* (2001) as an SVM extension. It was designed for anomaly or novel detection and has been widely applied in bioinformatics (Bounsiar and Madden 2014). The RBF kernel is usually introduced to OCSVM as a kernel function to measure the similarity in a high-dimension space, and the RBF kernel-based OCSVM model is adept at identifying the “small” group of outliers out of a large candidate pool. As our dataset was generated from compound libraries with *de novo* structures, no experimentally validated biological activities could be labeled to data points and serve as ground truth for model training. In response, we formulated an automated parameter tuning OCSVM model, referred to as APT-OCSVM, to conduct unsupervised learning with the entire post-selection dataset and allow automated positive detection in absence of pre-labeled training data. The APT-OCSVM is a hybrid model combining *k*-means clustering (Chen *et al.* 2024) and the unsupervised similarity computing (Ogbuabor and Ugwoke 2018) that could automatically calculate the optimal ν (nu) and enhance hit detection with the most promising outputs from the complex dataset. Here, we applied this unsupervised classification model to our dataset of 30 million entries. In APT-OCSVM, the input compounds were labeled as “negative” or “positive” (Fig. 3a, available as supplementary data at *Bioinformatics* online), with the 10%–20% negative candidates filtered out automatically while positive-classified candidates retained for subsequent processing.

### 3.5 A level 1 algorithm predicted the most proactive compounds

After the sequential filtering from descriptor definition to OCSVM classification, a vast majority of background signals have been efficiently excluded from noisy post-selection DEL datasets, with a small class of potential positives identified out of the 30.42 million-membered library. However, there were still ∼13 000 compounds remained (Table 2a, available as supplementary data at *Bioinformatics* online), which was too distracting to determine the most promising ones. Therefore, the AI algorithmic filtering was iterated to finalize the hit candidates before activity prediction. In level 0 filtering algorithm, the input dataset was denoised and grouped into negative and positive candidates in APT-OCSVM, and only positive candidates were proceeded into level 1 algorithm (Fig. 3a and Fig. 1, available as supplementary data at *Bioinformatics* online). Then, the data underwent the same filtering workflow from descriptor definition, normalization, BDF to DBSCAN. As a result, 2027 compounds entered OCSVM, of which 643 were classified as higher priority candidates over other structures this time (Fig. 3b and Table 2b, available as supplementary data at *Bioinformatics* online). In level 1 algorithm, OCSVM only served as classification method without filtering out any “negative” candidates. We reason that, negative backgrounds have been expelled in level 0 algorithm, while low-confidence molecules removed after DBSCAN in level 1 algorithm. Therefore, we retained all 2027 compounds as hit candidates to be imported to subsequent IPCA clustering and similarity analysis.

Here, Shapley values were utilized to identify the most significant features in filtering positive candidates, thus elucidating how OCSVM predicted compound classes during its learning process and estimating the probability of predicted active compounds by OCSVM (Mastropietro *et al.* 2023). As multiple descriptors were incorporated in OCSVM classification, we proposed to evaluate their importance on detecting the most promising compound from the positive candidates in level 1 algorithm. Shapley additive explanations (SHAP) were used to estimate Shapley values for involved features across all possible coalitions, and the descriptors were ordered in accordance with their contribution to the predicted probabilities (Fig. 3b). It was evident that $S_{c}$, $\sigma_{C}$, $\sigma_{R}$, and $S_{R}$ were more significant than $S_{nR}$ and $S_{nC}$ in identifying the most potential candidates, while the latter contributed more to detecting possibly active candidates. As illustrated with a sample coalition, $S_{nR}$, $\sigma_{R}$, $\sigma_{C}$, $S_{c}$, and $S_{R}$ collectively forced the SHAP values up to approximately 0.6, indicating that the predicted probability of the compound being active was around 60%. Conversely, the feature $S_{nc}$ exerted a downward pressure on the SHAP value (Fig. 3c). Furthermore, the dependency was revealed for the SHAP value of $S_{R}$ on the count of non-zero richness within our dataset (Fig. 3d), where $S_{R}$ and $S_{nR}$ exhibited greater contribution to SHAP when SHAP-transformed $S_{R}$ values located within 0–1. Lower values of $S_{R}$ and $S_{nR}$ were associated with lower predicted SHAP values; however, as the dependency revealed a non-linear correlation of SHAP to the two variables within 0–1 transformed $S_{R}$ values, increasing $S_{R}$ alone might drive a higher predicted SHAP value even $S_{nR}$ remained unchanged. In contrast, when transformed $S_{R}$ values were larger than 1, SHAP would present a linear correlation to the two variables.

### 3.6 IPCA facilitated data visualization and hit candidate clustering

The positive compounds have been well differentiated from statistically confidence datasets with OCSVM; however, multiple dimensions, including 10 features and one predicted label, were incorporated to characterize one compound in the algorithm, making data visualization rather intricate. Thus, it was still required to simplify the descriptive variables for straightforward data presentation. In this regard, PCA can serve as an effective method to enable visualization of the high-dimensional data in lower-dimensional space. This unsupervised machine learning algorithm was first proposed by Pearson (1901) and later developed by Oja (1982) and Sanger (1988) to 1D and hierarchical multidimensional PCA, respectively. The PCA technique has been widely implemented to cheminformatics analysis, while Benigni *et al.* further introduced PCA as one of the quantitative modeling techniques for analyzing multivariate data in biological research (Benigni and Giuliani 1994). PCA realizes dimensionality reduction while preserving essential information by defining principal components with maximum variance, thereby generating 2D and 3D scatter graphs that clearly show the locations of active and inactive compounds. As DEL selections yielded in a rather larger set of data, IPCA, a variant of PCA that is particularly effective for large datasets, was employed to visualize the OCSVM results in both level 0 and level 1 algorithms.

Notably, clustering for hit candidates could also be achieved accompanying data visualization in IPCA (Fig. 3e and f). Multiple characteristic features were reduced to a single dimension with anti-phosphorylated INSR and anti-INSR selections projected to *y*- and *x*-direction, respectively, indicating that the hit candidates were positioned according to their binding performance toward activated or basal INSR. Thus, the hit candidates were sorted into positive and negative clusters (right and left to the central axis, respectively) spontaneously during IPCA visualization. The anti-pY1355 and anti-pY1361 to anti-INSR cluster visualization were presented, respectively, where the positive overlaps would be regarded as effective candidates. In our previous study (Huang *et al.* 2024), compounds **134-207-192**, **191-245-192**, and **235-245-192** were experimentally validated to be agonists that could induce the intracellular phosphorylation and activation of INSR, while compound **52-194-192** that were clustered as negatives in anti-pY1361 visualization did not exhibit activating activities on INSR, which corroborated our data analysis results.

### 3.7 Similarity analysis with INSR metric enhancement facilitates accurate hit prediction

To identify the most promising hit compounds, the candidates were scored and ranked according to their performance across multiple selection replicates using IPCA computation, in aim of predicting corresponding bioactivities on the targeted protein INSR. As agonists are likely to exhibit higher affinity against activated INSR over basal INSR, it could be anticipated that the potential hit compound should give a higher enrichment on phosphorylated INSR than total INSR. To further enhance the processing robustness on biasing the method output toward agonistic hits, we proposed two more metrics, namely INSR comparison indicator and conditional summation, respectively. The INSR comparison indicator was summed up based on ligand binding preference toward phosphorylated INSR or total INSR across the selection replicates and were utilized in similarity analysis for calculating DEL candidate scores. The introduction of the INSR comparison indicator would be able to greatly enhance the ranking accuracy.

Besides, we proposed a conditional calculation instead of simply summing the richness values and abundance count across all replicates. For each compound, the five replicates were grouped based on their performance on phosphorylated INSR relative to total INSR, and only the dominant group (containing over 50% of total replicates) would be retained and corresponding metrics be summed. Accordingly, if a compound demonstrated superior performance against pY1355/pY1361 or INSR in the majority of the replicates, this preference contrast would be strengthened by reducing the influence of outliers and ensuring that minority deviations would not disproportionately affect overall performance metrics. This conditional calculation was incorporated in aim of mitigating the impact of potential biases that arose from inconsistent data quality across individual folds.

To demonstrate the performance of the two different strategies, comparative study was carried out with richness display as well as IPCA similarity analysis. We applied the two distinct methods to both richness and abundance count in the 30.42-million DEL dataset: the simple summation across all data files and our conditional calculation across dominant replicates. The first method provided a general overview of DEL candidates across all involved targets without considering specific conditions. However, in practice, the quality of collections could vary significantly across different environments, leading to inconsistent performance in individual folds. This variability could skew the overall results when using this method. In contrast, the latter method addresses this bias by focusing on the binding performance from the majority of replicates.

**Figure 4:**
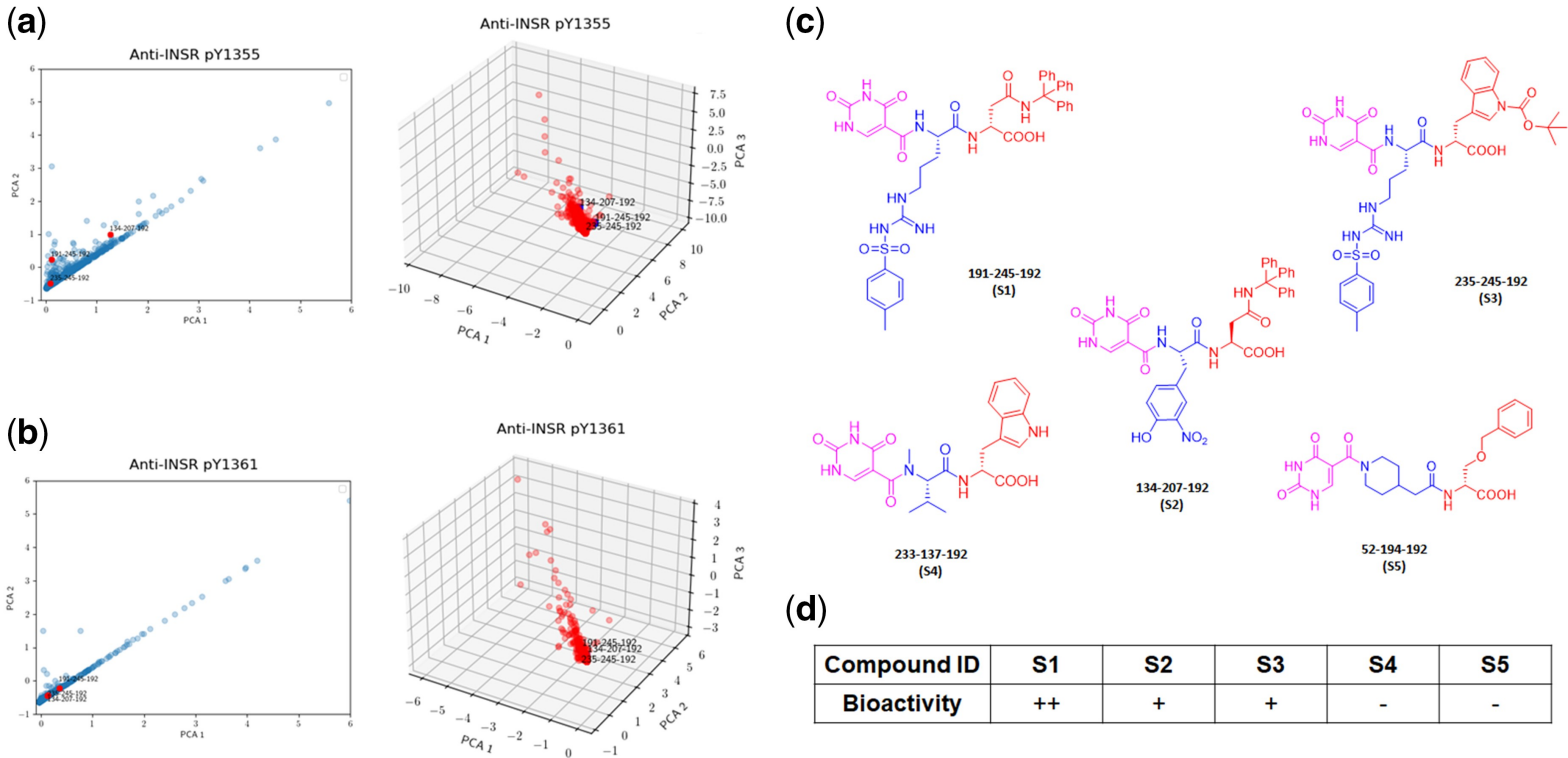
Similarity analysis accurately predicted hit compounds from the 30.42 million DEL selections against INSR on live cells. (a) IPCA visualization based on anti-pY1355 data against anti-INSR data after similarity analysis with INSR indicator enhancement. (b) IPCA visualization based on anti-pY1361 data against anti-INSR data after similarity analysis with INSR indicator enhancement. Left: 2D visualization; right: 3D visualization. (c) Structures of the hit compounds. (d) Bioactivity readouts after experimental hit validation. ++: high bioactivity; +: moderate bioactivity; −: no bioactivity.

The improvement was first demonstrated with richness display for straightforward comparison of the two strategies. As illustrated in Fig. 4, available as supplementary data at *Bioinformatics* online, an example was presented using a cherry-picked compound **186-5-89**, and the compound’s richness in pY1355 and INSR files was listed to indicate its binding performance across separate replicates. In the first method, the simple summation of richness across all pY1355 and INSR files was conducted. A lower overall richness in anti-pY1355 compared to anti-INSR was disclosed with the compound positioned in the lower right of the richness diagram, suggesting that compound **186-5-89** performed weaker on pY1355 than on total INSR. This conclusion was heavily influenced by the exceptionally high richness value of 3153.48 in the 12-INSR.erh file compared to 2.35 in the 10-pY1355.erh file, which distorted the overall anti-INSR performance assessment when using the first method. On the other hand, our method mitigated such biases by prioritizing consistency across multiple replicates. Since compound **186-5-89** performed better in pY1355 files in over 50% of experimental groups (1-pY1355.erh, 7-pY1355.erh, and 13-pY1355.erh, three out of five), only the richness values from the three replicates were summed. The total richness in pY1355 was calculated as 1628.53, which exceeded the richness in INSR of 8.85. The richness pattern was also tuned after conditional summation, where **186-5-89** was located at upper right region. These indicated that the compound was more active in pY1355 than in INSR. Thus, our method could reduce the biased impact of a single fold’s performance, providing a more accurate reflection of its overall performance.

**Figure 5.**
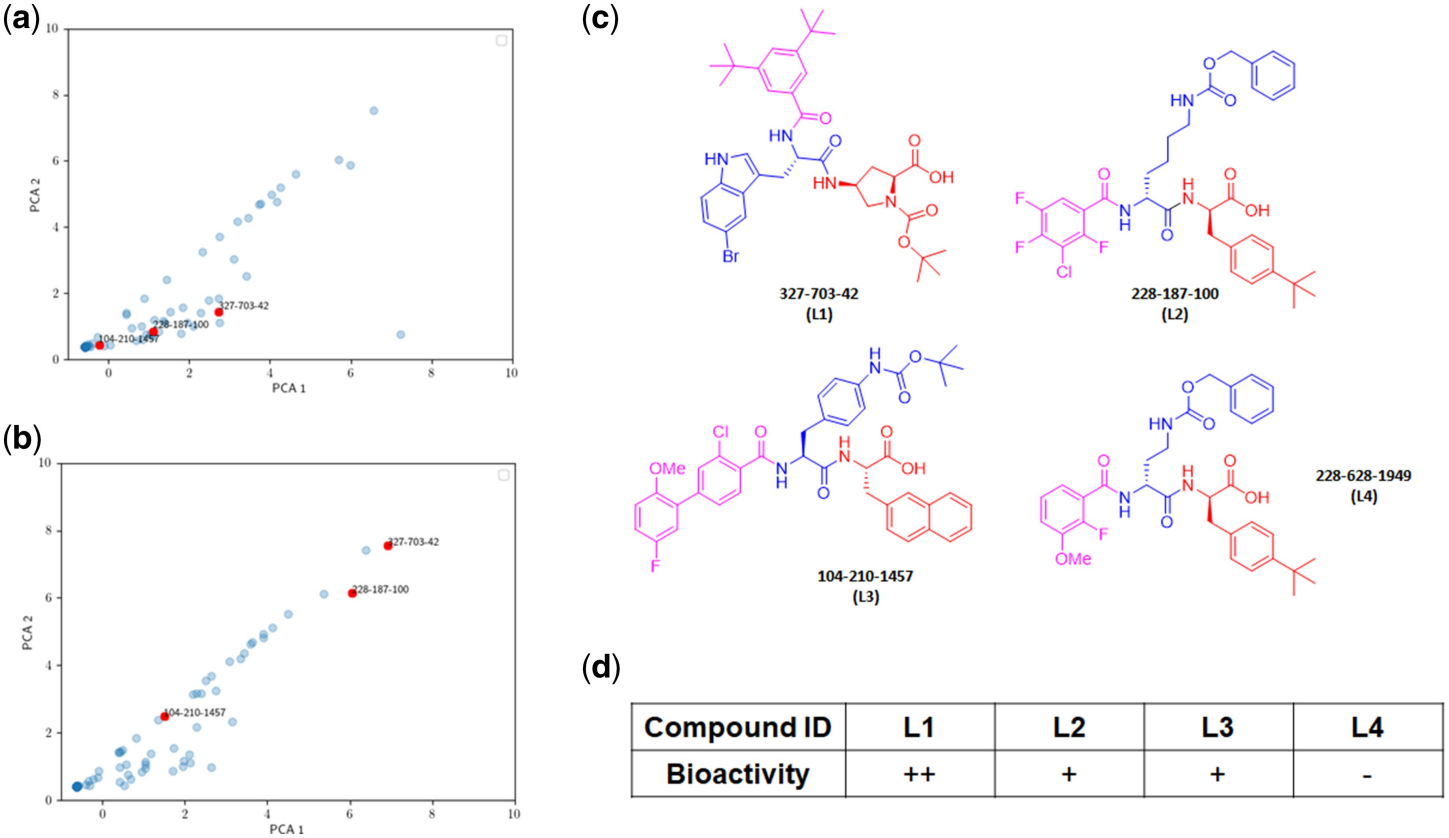
Similarity analysis predicted hit compounds from the 1.033 billion DEL selections against INSR on live cells. (a) IPCA visualization based on anti-pY1355 data against anti-INSR data after similarity analysis with INSR indicator enhancement. (b) IPCA visualization based on anti-pY1361 data against anti-INSR data after similarity analysis with INSR indicator enhancement. (c) Structures of the hit compounds. (d) Bioactivity readouts after experimental hit validation. ++: high bioactivity; +: moderate bioactivity; −: no bioactivity.

In our method, the most proactive compounds enhanced by the binding preference were clearly presented along the upper right edge of the hyperplane of the clusters after similarity analysis (Fig. 4a and b), with the ranking scores obtained simultaneously (Table 5, available as supplementary data at *Bioinformatics* online). Indeed, compounds **191-245-192** (S1, available as supplementary data at *Bioinformatics* online), **134-207-192** (S2, available as supplementary data at *Bioinformatics* online), and **235-245-192** (S3, available as supplementary data at *Bioinformatics* online) ranked at 7, 19, and 439 (Fig. 4c) showed strong agonistic activities on INSR in our previous study (Huang *et al.* 2024), whereas compound **233-137-192** (S4, available as supplementary data at *Bioinformatics* online) that was removed during BDF in level 1 algorithm and **52-194-192** (S5, available as supplementary data at *Bioinformatics* online) that was clustered negative in anti-pY1361 (Fig. 3e and f) showed no activity (Fig. 4d). Moreover, our earlier work also showed that compound S1 had the highest activity on INSR activation (Huang *et al.* 2024), in agreement with algorithmic prediction where it was ranked top among the three validated molecules (Table 5, available as supplementary data at *Bioinformatics* online). The high consistency has demonstrated the reliability of our AI filtering approach in facilitating hit determination from highly noisy cell-based DEL selections. In contrast, compounds S1 and S2 were positioned at left region to the hyperplane in anti-pY1355 and S3 in anti-pY1361 after similarity analysis without INSR indicator enhancement (Fig. 5, available as supplementary data at *Bioinformatics* online), indicating a superior performance with the conditional computation.

### 3.8 AI filtering enables agonist identification from a 1.033 billion-membered DEL

Subsequent to methodology establishment with the 30.42 million-membered DEL dataset, we also tested this AI-filtering algorithm with a larger DEL containing ∼1.033 billion molecules. This larger-scale DEL also underwent selection by cell-based INSR and the data outcome was subjected to a same sequential filtering process in the dual-level algorithm to exclude the vast majority of the noisy signals (Figs 6 and 7, available as supplementary data at *Bioinformatics* online). The Shapley Value Analysis has revealed a similar contribution pattern of OCSVM features to the 30.42 million DEL dataset (Fig. 8, available as supplementary data at *Bioinformatics* online). Particularly, a beeswarm plot was used to illustrate the entire distribution of SHAP values for involved features. As depicted, $S_{c}$ is the most significant contributor in global coalitions, indicating that a higher $S_{c}$ value was associated with a greater probability to be classified as positive; while $S_{R}$and $\sigma_{R}$ played more crucial roles in local collations for binary classification predictions, implying their substantial magnitude impact on the average individual cases (Fig. 8c, available as supplementary data at *Bioinformatics* online).

IPCA clustering and similarity analysis have predicted agonistic activities in visualization for positive candidates in the 1.033 billion DEL (Fig. 5a and b and Table 3, available as supplementary data at *Bioinformatics* online) and score-ranked the most proactive compounds (Table 6, available as supplementary data at *Bioinformatics* online). The compounds clustered positive and ranked top at 1, 2, and 18 were decoded as **327-703-42** (L1), **228-187-100** (L2), and **104-210-1457** (L3), respectively (Fig. 5c). Again, the previous study showed that L1 could trigger a significant activating response from cellular INSR, while L2 and L3 had a moderate phosphorylation effect on INSR pY1355/1361 (Fig. 5d) (Huang *et al.* 2024). The activity orders coordinated well with the predicted ones, further enhancing the confidence of our filtering algorithm. Compound **228-628-1949** (L4), which was categorized as negative in IPCA clustering of anti-pY1355 against anti-INSR in level 1 algorithm (Fig. 9, available as supplementary data at *Bioinformatics* online), also appeared to be non-agonists on INSR in our previous experiments (Huang *et al.* 2024). The results have suggested a satisfactory accuracy rate on predicting agonistic molecules from varied dataset scales.

### 3.9 Automated filtering could be effectively applied to other protein targets

To further demonstrate algorithmic performance on protein targets beyond INSR, we proposed to apply it to another membrane protein TPOR. The TPOR-derived dataset was generated from the 30.42-million DEL selections (Huang *et al.* 2024). Briefly, the library molecules were visited by on-cell TPOR, with binding entities recovered after parallel pulldown by anti-TPOR pY626 and anti-TPOR antibodies, respectively. The post-selection DELs were transformed to sequencing data and processed with the developed filtering pipeline. Specially, an analogous TPOR comparison indicator was incorporated corresponding to INSR comparison indicator to enhance similarity analysis.

After the dual-level filtering, 130 compounds were retained as proactive hit candidates, of which 38 were categorized as the most promising ones (Fig. 12a and b and Table 7, available as supplementary data at *Bioinformatics* online). The compounds predicted to be 1 and 7 in similarity ranking turned out to be **124-159-422** (M1) and **57-143-358** (M2) that have been previously validated to be agonists for TPOR (Fig. 12c and d and Table 8, available as supplementary data at *Bioinformatics* online) (Huang *et al.* 2024), signifying that this approach could also be effectively applied to cellular targets other than INSR. Collectively, our automated AI-filtering method has demonstrated superb hit prediction performance, and that it is generally applicable to cell-based selections with different sized DELs as well as diverse target proteins, provided that they share a similar design logic.

## 4 Conclusion and discussion

We advanced an AI framework hybridizing automated outlier filtering with unsupervised classification to isolate active compounds targeting protein of interest (POI) from DELs dominated by inactive entries. Our method integrates the Selection Performance Descriptors, standardized input pre-processing, and an AEC-optimized DBSCAN algorithm for outlier removal. An OCSVM classifier then separates active from inactive DEL members. The framework operates at two levels: level 0 identifies all potential candidates, while level 1 refines these to prioritize the most potential hits. This hierarchical approach aims to maximize hit accuracy and uncover bioactive compounds within vast unlabeled datasets, overcoming the constraints of conventional ML strategies. By bridging the gap between supervised and unsupervised paradigms, our work advances the precision of DEL-based drug discovery and offers a scalable solution for high-noise, imbalanced and unlabeled data environments.

A key innovation lies in the self-adaptability across the algorithm workflow, where the parameters could be tuned automatically responding to different data features. This unsupervised algorithm is capable of removing the noises and filtering out the less proactive compounds from large unlabeled datasets. In principle, this AI filtering may be implemented with all types of DEL datasets with analogous hit picking logics. An instance for automation is the integration of a self-adaptive mechanism that automates critical DBSCAN parameters—specifically, the *ε* (epsilon) radius and MinPts (minimum points to construct a dense region) threshold—ensuring optimal cluster definition. This adaptive architecture dynamically recalibrates parameters in response to variations in input dataset features, enabling consistent outlier detection across heterogeneous descriptor spaces. Consequently, the proposed method not only refines descriptor quality by removing noise but also enhances dataset robustness, offering a scalable solution for high-throughput DEL screening. Another major improvement lies in the introduction of bias enhancement factors as candidate scoring and ranking metrics. The binding performance on phosphorylated target over total target, featured by INSR comparison indicator and conditional summation, is incorporated into similarity analysis as benchmark metrics to prioritize the bias contribution, thus enhancing hit prediction accuracy from DEL selection dataset with certain binding bias. Notably, we have successfully extended this filtering scheme to a much larger DEL covering 1.033 billion molecular structures and another cellular target TPOR, implying that it is a capable method for diverse DELs and various targeted proteins.

In experimental bioactivity validation, only limited compounds among the top ranked candidates were previously synthesized and tested their agonism on cellular INSR (Huang *et al.* 2024) and the practical hit rate of this hybrid AI framework has yet to be determined. We anticipate performing hit rate analysis by covering more top ranked molecules in biological activity validation, thus acquiring a more comprehensive accuracy evaluation. Besides, we propose to set up supervised neural network or large models based on accumulated bioactivity information so as to predict active compounds with higher accuracy.

It should be noted that the AI framework was specially designed to differentiate agonists from binders and other background signals. Hence, the selection logic laid on the observation that the agonist is to be enriched in both phosphorylated and total targets only that the phosphorylated target will yield a higher agonist recovery. However, in most cases the DEL selections are performed in the target-sufficient versus target-deficient manner, which required a disparate hit identification logic. As a result, the implementation is currently limited to agonist discovery. In response, we also aim to further incorporate other commonly involved DEL data processing logics in the future work to expand the application scenarios for our method.

## Supplementary Material

btag001_Supplementary_Data

## Data Availability

The data underlying this article are available in DEL_AI: DEL_AI V0.0, at https://dx.doi.org/10.5281/zenodo.17452392 and INSR and TPOR RS and ERH files, at https://dx.doi.org/10.5281/zenodo.17569557.
