# Supplementary material for: A hybrid unsupervised methodology on artificial intelligence filtering for automatically processing cellular DNA-encoded library (DEL) datasets
Source: Bioinformatics. 2026 Jan 7;42(1):btag001. doi: 10.1093/bioinformatics/btag001 (PMC12836421; doi:10.1093/bioinformatics/btag001)
Supplement: btag001_Supplementary_Data [file btag001_supplementary_data.docx]

**Supplementary Information**

**A Hybrid Unsupervised Methodology on Artificial Intelligence Filtering for automatically processing cellular DNA-Encoded Library (DEL) Datasets**

Yiran Huang,^1*†^ Xiao Tan,^2*^ Xiaoyu Li,^3,4^ Feng Xiong,^3,5^  and Siu Ming Yiu^2†^

^1^ School of Pharmacy, Shenzhen University Medical School, Shenzhen University, Shenzhen 518060, China

^2^ Department of Computer Science, The University of Hong Kong, Pokfulam Road, Hong Kong SAR 999077, China

^3^ Department of Chemistry and State Key Laboratory of Synthetic Chemistry, The University of Hong Kong, Pokfulam Road, Hong Kong SAR 999077, China

^4^ Laboratory for Synthetic Chemistry and Chemical Biology Limited, Health@InnoHK, Innovation and Technology Commission, Units 1503-1511, 15/F., Building 17W, Hong Kong SAR 999077, China

^5^ Current address: Shenzhen NewDEL Biotech Co., Ltd., Shenzhen 518110, China

^*^ These authors contributed equally: Yiran Huang, Xiao Tan

Corresponding Author

^†^ huangyiran@szu.edu.cn; smyiu@cs.hku.hk

# **Supplementary figures and tables referenced in the main text.**

## **Table S1. Selection Performance Descriptors in terms of richness (**$\boldsymbol{R}$**) and abundance count (**$\boldsymbol{C}$**) for each DEL member against target or phosphorylated target.**

| **ID** | **Type of Tiers** | **Symbol** | **Formula** | **Statistical Descriptors** |
| --- | --- | --- | --- | --- |
| 1 | Tier 2 | $S_{R}$ | $S_{R}=\sum R_{i}$  $S_{R}$: Sum of richness, $R_{i}$ is the richness of a DEL compound in repeat$i$. | Effectiveness Indicator |
| 2 | Tier 2 | $S_{C}$ | $S_{C}=\sum C_{i}$  $S_{C}$: Sum of abundance count, $C_{i}$ is the abundance count of a DEL compound in repeat$i$ | Effectiveness Indicator |
| 3 | Tier 2 | $S_{nR}$ | $S_{nR}=\sum{nR}_{i}$  $S_{nR}$: Sum of richness reproducibility, ${nR}_{i}$describes whether a DEL compound is enriched in repeat$i$. It is 1 if the richness is higher than 1, and is 0 if the richness is lower than 1. | Reproducibility Indicator |
| 4 | Tier 2 | $S_{nC}$ | $S_{nC}=\sum{nC}_{i}$  $S_{nC}$: Sum of abundance count reproducibility, ${nC}_{i}$describes whether a DEL compound occurs in repeat$i$. It is 1 if the abundance count is not 0, and is 0 if the abundance count is 0. | Reproducibility Indicator |
| 5 | Tier 2 | $\sigma_{R}$ | $\sigma_{R}=\sqrt{\frac{\sum(R_{i}-\bar{R} )}{N}}$  $\sigma_{R}$: Standard deviation of richness for a DEL compound, $N$is total number of repeats in the calculated dataset. | Stability Indicator |
| 6 | Tier 2 | $\sigma_{C}$ | $\sigma_{C}=\sqrt{\frac{\sum(C_{i}-\bar{C} )}{N}}$  $\sigma_{C}$: Standard deviation of the abundance count for a DEL compound, $N$is the total number of repeats in the calculated dataset. | Stability Indicator |
| 7 | Tier 3 | $\Phi_{eff}$ | $\Phi_{eff}=\frac{\sum R_{i}}{\sum C_{i}}$  $\Phi_{eff}$: Average richness over abundance count among repeats. | Effectiveness Indicator |
| 8 | Tier 3 | $K_{R}$ | $K_{R}=\frac{\sum R_{i}}{\sqrt{\frac{\sum(R_{i}-\bar{R)}}{N}}}$  $K_{R}$: Average richness over standard deviation among repeats. | Balance Indicator |
| 9 | Tier 3 | $K_{C}$ | $K_{C}=\frac{\sum C_{i}}{\sqrt{\frac{\sum(C_{i}-\bar{C)}}{N}}}$  $K_{C}$: Average abundance count over standard deviation among repeats. | Balance Indicator |
| 10 | Tier 3 | $\Phi_{bal}$ | $\Phi_{bal}=\left\vert log(\frac{\frac{\sum R_{i}}{\sqrt{\frac{\sum(R_{i}-\bar{R)}}{N}}}}{\frac{\sum C_{i}}{\sqrt{\frac{\sum(C_{i}-\bar{C)}}{N}}}}) \right\vert$  $\Phi_{bal}$: Absolute value of logarithm of the richness stability to the abundance count stability. | Balance Indicator |

## **Table S2. Overview of AI filtering results for 30.42 million-membered DEL on INSR.**

**a) Level 0 algorithm**

| **Steps** | **DEL Processing** | **Stepwise Output** | **Filter In (%)** | **Filter Out (%)** |
| --- | --- | --- | --- | --- |
| **1** | **Input** | 30,487,600 | - | - |
| **2** | **Pre-processing** | 1,004,229 | 3.29% | 96.71% |
| **3** | **BDF** | 16,067 | 1.60% | 98.40% |
| **4** | **AEC-based DBSCAN** | 16,065 | 99.99% | 0.01% |
| 5 | INSR metric enhancement | 16,065 | 100% | 100% |
| **6** | **RBF kernel-based OCSVM** | 12,852 | 80.00% | 20.00% |
| 7 | IPCA visualization | 12,852 | 100% | 100% |
| 8 | Similarity analysis | 12,852 | 100% | 100% |

**b) Level 1 algorithm**

| **Steps** | **DEL Processing** | **Stepwise Output** | **Filter In (%)** | **Filter Out (%)** |
| --- | --- | --- | --- | --- |
| **9** | **Input** | 12,852 | - | - |
| **10** | **Pre-processing** | 12,818 | 99.74% | 0.26% |
| **11** | **BDF** | 2,028 | 15.82% | 84.18% |
| **12** | **AEC-based DBSCAN** | 2,027 | 99.95% | 0.05% |
| **13** | **INSR metric enhancement** | 2,027 | 100% | 0% |
| **14** | **RBF kernel-based OCSVM** | 2,027 | 100% | 0% |
| **15** | **IPCA clustering** | 2,027 | 100% | 0% |
| **16** | **Similarity analysis** | 643 | 31.72% | 68.28% |

## **Table S3. Overview of AI filtering results for 1.033 billion-membered DEL on INSR.**

**a) Level 0 algorithm**

| **Steps** | **DEL Processing** | **Stepwise Output** | **Filter In (%)** | **Filter Out (%)** |
| --- | --- | --- | --- | --- |
| **1** | **Input** | 1,032,495,408 | - | - |
| **2** | **Pre-processing** | 7,941,679 | 14.09% | 85.91% |
| **3** | **BDF** | 127,066 | 0.06% | 99.94% |
| **4** | **AEC-based DBSCAN** | 127,066 | 100% | 0% |
| 5 | INSR metric enhancement | 127,066 | 100% | 100% |
| **6** | **RBF kernel-based OCSVM** | 101,653 | 88.91% | 11.09% |
| 7 | IPCA visualization | 101,653 | 100% | 100% |
| 8 | Similarity analysis | 101,653 | 100% | 100% |

**b) Level 1 algorithm**

| **Steps** | **DEL Processing** | **Stepwise Output** | **Filter In (%)** | **Filter Out (%)** |
| --- | --- | --- | --- | --- |
| **9** | **Input** | 101,653 | - | - |
| **10** | **Pre-processing** | 101,653 | 100% | 0% |
| **11** | **BDF** | 3,200 | 3.15% | 96.85% |
| **12** | **AEC-based DBSCAN** | 3,200 | 100% | 0% |
| **13** | **INSR metric enhancement** | 3,200 | 100% | 0% |
| **14** | **RBF kernel-based OCSVM** | 3,200 | 100% | 0% |
| **15** | **IPCA clustering** | 3,200 | 100% | 0% |
| **16** | **Similarity analysis** | 208 | 6.5% | 93.5% |

## **Table S4. Example comparison of conditional summation with simple summation.**

**a) Compound 186-5-89 in 30.42 million-membered DEL**

| **Anti-pY1355 Selection** | **Richness on INSR pY1355** | **Anti-INSR Selection** | **Richness on total INSR** | **Binding Preference** |
| --- | --- | --- | --- | --- |
| **1-pY1355.erh** | 309.35 | 3-INSR.erh | 3.29 | pY1355>INSR |
| **4-pY1355.erh** | 0 | 6-INSR.erh | 5.56 | pY1355<INSR |
| **7-pY1355.erh** | 1306.95 | 9-INSR.erh | 0 | pY1355>INSR |
| **10-pY1355.erh** | 2.35 | 12-INSR.erh | 3153.48 | pY1355<INSR |
| **13-pY1355.erh** | 12.23 | 15-INSR.erh | 0 | pY1355>INSR |
| **Simple Summation** | 1630.88 | < | 3162.33 | pY1355<INSR |
| **Conditional Summation** | 1628.53 | > | 8.85 | pY1355>INSR |

**b) Compound 104-210-1457 in 1.033 billion-membered DEL**

| **Anti-pY1355 Selection** | **Richness on INSR pY1355** | **Anti-INSR Selection** | **Richness on total INSR** | **Binding Preference** |
| --- | --- | --- | --- | --- |
| **1-pY1355.erh** | 22.7 | 3-INSR.erh | 15.03 | pY1355>INSR |
| **4-pY1355.erh** | 295.24 | 6-INSR.erh | 98.1 | pY1355>INSR |
| **7-pY1355.erh** | 0 | 9-INSR.erh | 2365.08 | pY1355<INSR |
| **10-pY1355.erh** | 314.29 | 12-INSR.erh | 123.17 | pY1355>INSR |
| **13-pY1355.erh** | 36.98 | 15-INSR.erh | 82.54 | pY1355<INSR |
| **16-pY1355.erh** | 9.52 | 18-INSR.erh | 48.41 | pY1355<INSR |
| **Simple Summation** | 678.73 | < | 2732.33 | pY1355<INSR |
| **Conditional Summation** | 632.23 | > | 236.3 | pY1355>INSR |

## **Table S5. Predicted bioactivity ranking for 30.42 million-membered DEL on INSR**

| **Compound** | **Predicted Rank** | **Validated Activity** |
| --- | --- | --- |
| 132-216-192 | 1 | N.D. |
| 168-231-192 | 2 | N.D. |
| 237-42-192 | 3 | N.D. |
| 43-247-192 | 4 | N.D. |
| 148-233-192 | 5 | N.D. |
| 10-48-192 | 6 | N.D. |
| 191-245-192 | 7 | **Y** |
| 201-218-192 | 8 | N.D. |
| 29-226-70 | 9 | N.D. |
| 180-135-192 | 10 | N.D. |
| 222-162-192 | 11 | N.D. |
| 134-52-192 | 12 | N.D. |
| 14-197-140 | 13 | N.D. |
| 241-223-192 | 14 | N.D. |
| 175-165-385 | 15 | N.D. |
| 161-260-192 | 16 | N.D. |
| 91-154-233 | 17 | N.D. |
| 138-169-192 | 18 | N.D. |
| 134-207-192 | 19 | **Y** |
| 65-12-192 | 20 | N.D. |
| 235-245-192 | 439 | **Y** |

N.D.: Not detected as only some of the listed compounds were synthesized off-DNA and validated.

## **Table S6. Predicted bioactivity ranking for 1.033 billion-membered DEL on INSR**

| **Compound** | **Predicted Rank** | **Validated Activity** |
| --- | --- | --- |
| 327-703-42 | 1 | **Y** |
| 228-187-100 | 2 | **Y** |
| 299-703-1891 | 3 | N.D. |
| 576-601-1280 | 4 | N.D. |
| 315-703-1493 | 5 | N.D. |
| 550-480-1590 | 6 | N.D. |
| 672-621-1718 | 7 | N.D. |
| 367-587-588 | 8 | N.D. |
| 671-296-656 | 9 | N.D. |
| 624-653-1020 | 10 | N.D. |
| 688-81-776 | 11 | N.D. |
| 487-122-1679 | 12 | N.D. |
| 305-424-1642 | 13 | N.D. |
| 543-4-1138 | 14 | N.D. |
| 424-582-1071 | 15 | N.D. |
| 436-278-1222 | 16 | N.D. |
| 532-364-1435 | 17 | N.D. |
| 104-210-1457 | 18 | **Y** |
| 671-168-1873 | 19 | N.D |
| 23-213-502 | 20 | N.D. |

N.D.: Not detected as only some of the listed compounds were synthesized off-DNA and validated.

## **Table S7. Overview of AI filtering results for 30.42 million-membered DEL on TPOR.**

**a) Level 0 algorithm**

| **Steps** | **DEL Processing** | **Stepwise Output** | **Filter In (%)** | **Filter Out (%)** |
| --- | --- | --- | --- | --- |
| **1** | **Input** | 30,487,600 | - | - |
| **2** | **Pre-processing** | 60,832 | 0.20% | 99.80% |
| **3** | **BDF** | 973 | 1.60% | 98.40% |
| **4** | **AEC-based DBSCAN** | 972 | 99.90% | 0.108% |
| 5 | INSR metric enhancement | 972 | 100% | 0% |
| **6** | **RBF kernel-based OCSVM** | 874 | 89.92% | 10.08% |
| 7 | IPCA visualization | 874 | 100% | 0% |
| 8 | Similarity analysis | 874 | 100% | 0% |

**b) Level 1 algorithm**

| **Steps** | **DEL Processing** | **Stepwise Output** | **Filter In (%)** | **Filter Out (%)** |
| --- | --- | --- | --- | --- |
| **9** | **Input** | 874 | - | - |
| **10** | **Pre-processing** | 825 | 94.39% | 5.61% |
| **11** | **BDF** | 132 | 16.00% | 84.00% |
| **12** | **AEC-based DBSCAN** | 130 | 98.48% | 1.52% |
| **13** | **INSR metric enhancement** | 130 | 100% | 0% |
| **14** | **RBF kernel-based OCSVM** | 130 | 100% | 0% |
| **15** | **IPCA clustering** | 130 | 100% | 0% |
| **16** | **Similarity analysis** | 38 | 29.23% | 70.77% |

## **Table S8. Predicted bioactivity ranking for 30.42 million-membered DEL on TPOR**

| **Compound** | **Predicted Rank** | **Validated Activity** |
| --- | --- | --- |
| 124-159-422 | 1 | **Y** |
| 189-213-192 | 2 | N.D. |
| 63-257-205 | 3 | N.D. |
| 47-219-271 | 4 | N.D. |
| 37-229-192 | 5 | N.D. |
| 188-251-192 | 6 | N.D. |
| 57-143-358 | 7 | **Y** |
| 187-209-192 | 8 | N.D. |
| 178-13-205 | 9 | N.D. |
| 14-102-30 | 10 | N.D. |

N.D.: Not detected as only some of the listed compounds were synthesized off-DNA and validated.

## **Figure S1**


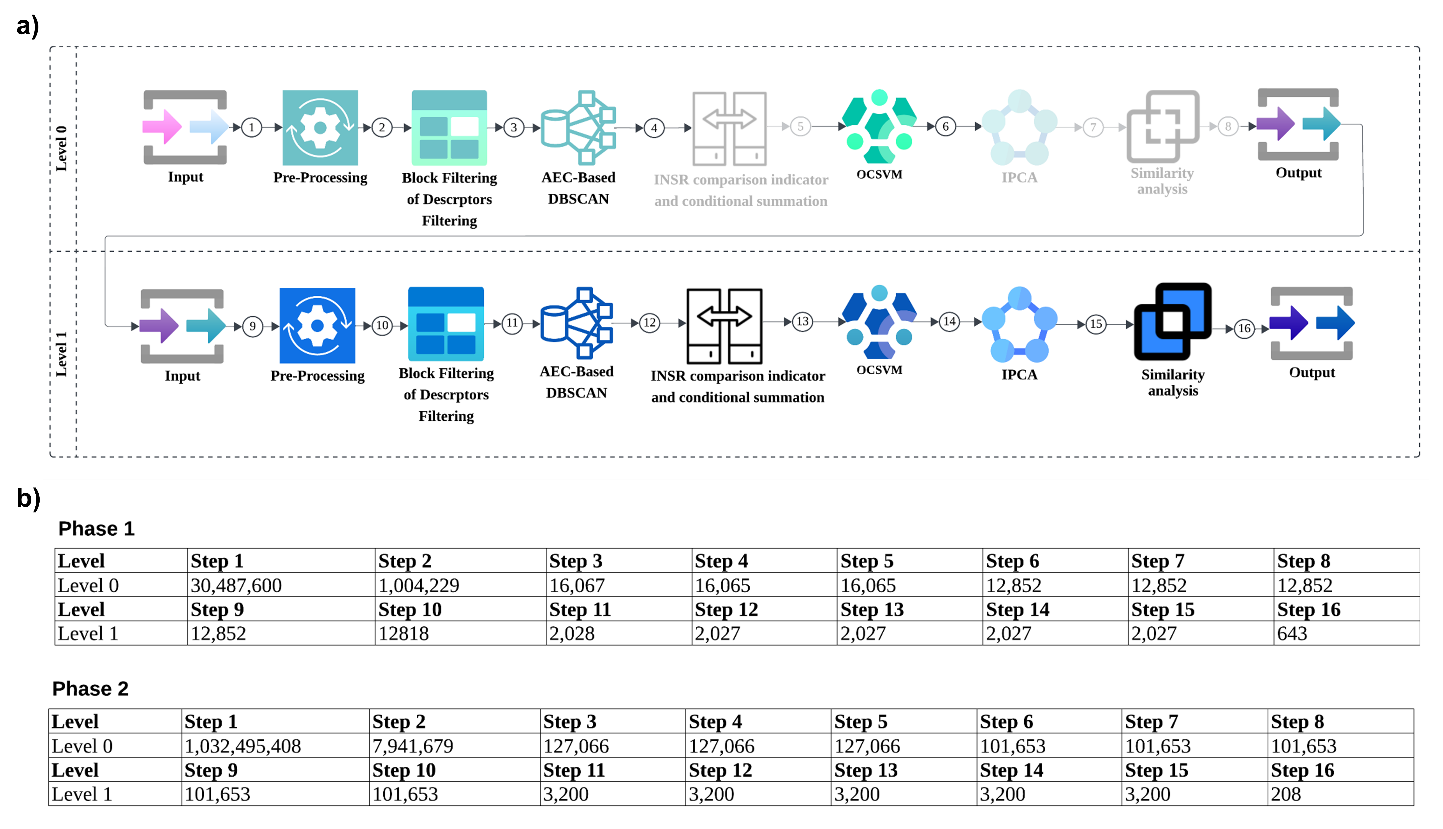


**Figure S1.** a) Detailed flowchart of the DEL-AI framework. The INSR metric enhancement, IPCA and similarity analysis in level 0 were shown as faded steps since they were incorporated to visualize and track progress in level 0 without execute filtering functions. b) Stepwise DEL compound outputs. See **Table S2** and **Table S3** for more details.

## **
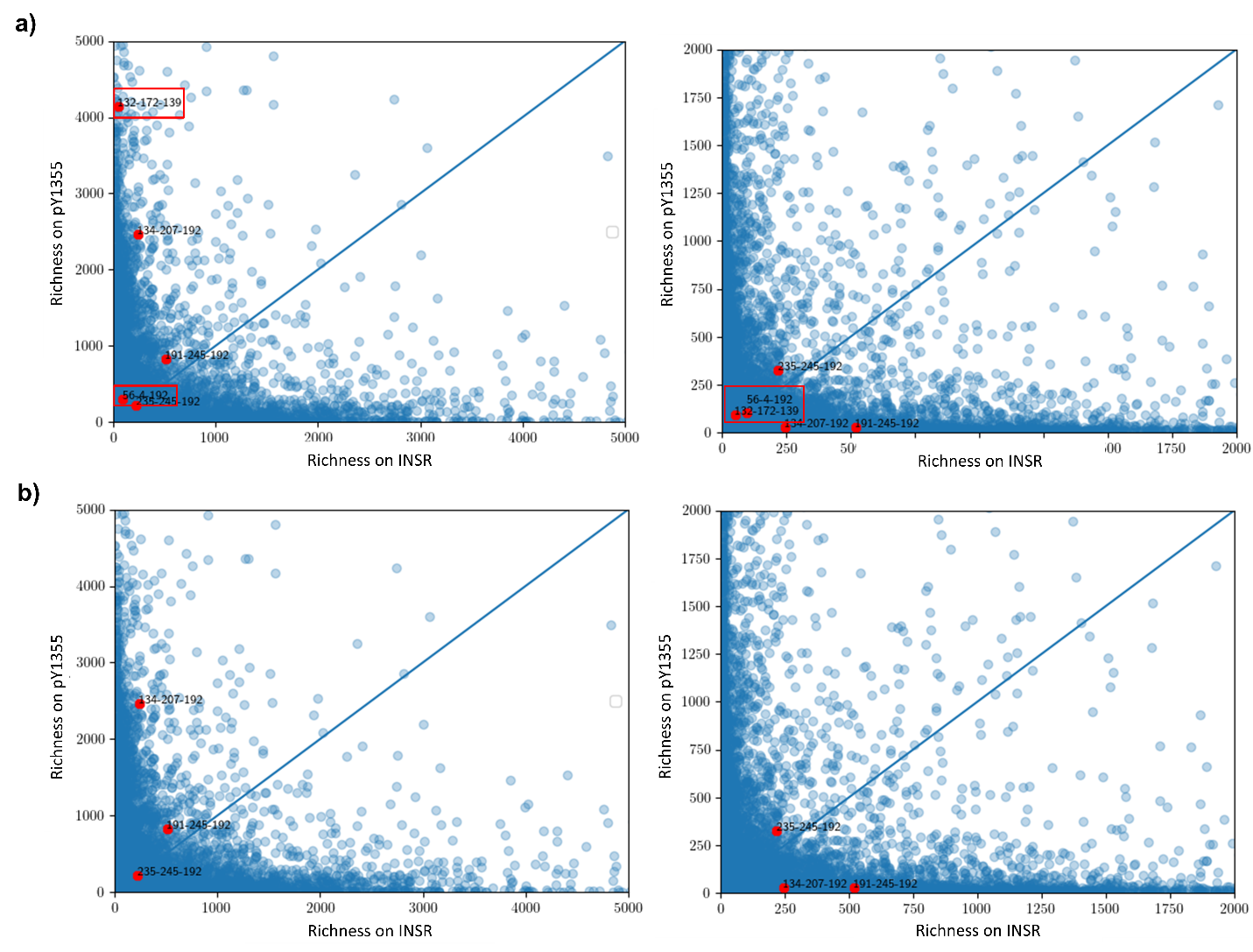
Figure S2**

**Figure S2.** AEC-based DBSCAN filtering for selection of a 30.42 million DEL against cellular INSR. a) Plot of the richness values of the selection results before DBSCAN filtering. Left: pY1355 to INSR; right: pY1361 to INSR. b) Plot of the richness values of the selection results after DBSCAN filtering. Left: pY1355 to INSR; right: pY1361 to INSR.

## **
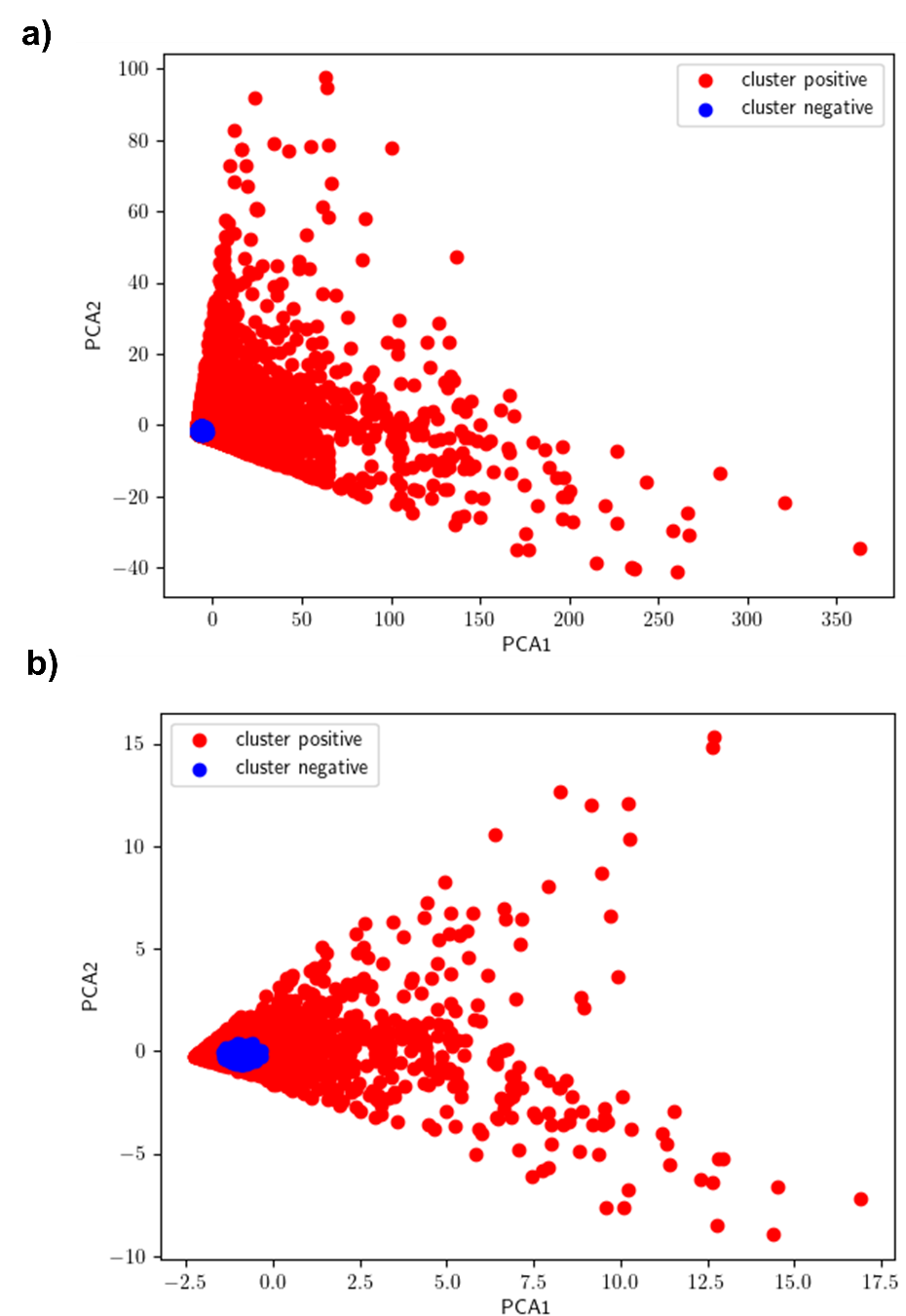
Figure S3**

**Figure S3.** RBF-kernel-based OCSVM classification for selection of the 30.42 million DEL against cellular INSR in a) level 0 algorithm and b) level 1 algorithm. The negative candidates are marked in blue, while the positive clusters are highlighted in red. The OCSVM results are visualized by IPCA. PCA1/PCA2: descriptive features regarding compound characteristics are IPCA-reduced to 2 dimensions.

##
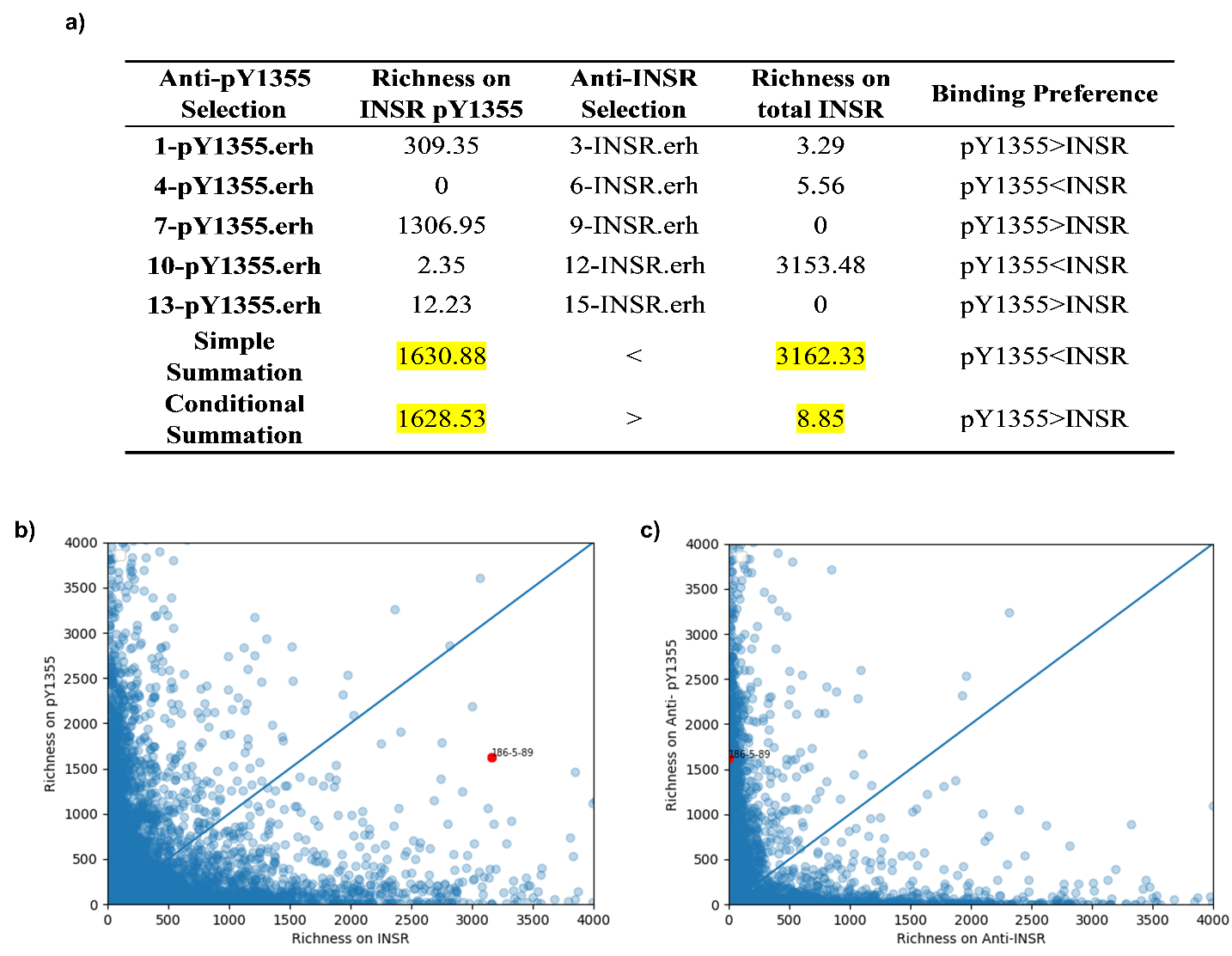
**Figure S4**

**Figure S4.** Comparison of simple summation with conditional summation with the 30.42 million DEL against cellular INSR. a) The performance of a presented example 186-5-89 in simple summation and conditional summation. b) Plot of the richness values of the selection results with simple summation. c) Plot of the richness values of the selection results with conditional summation.

##
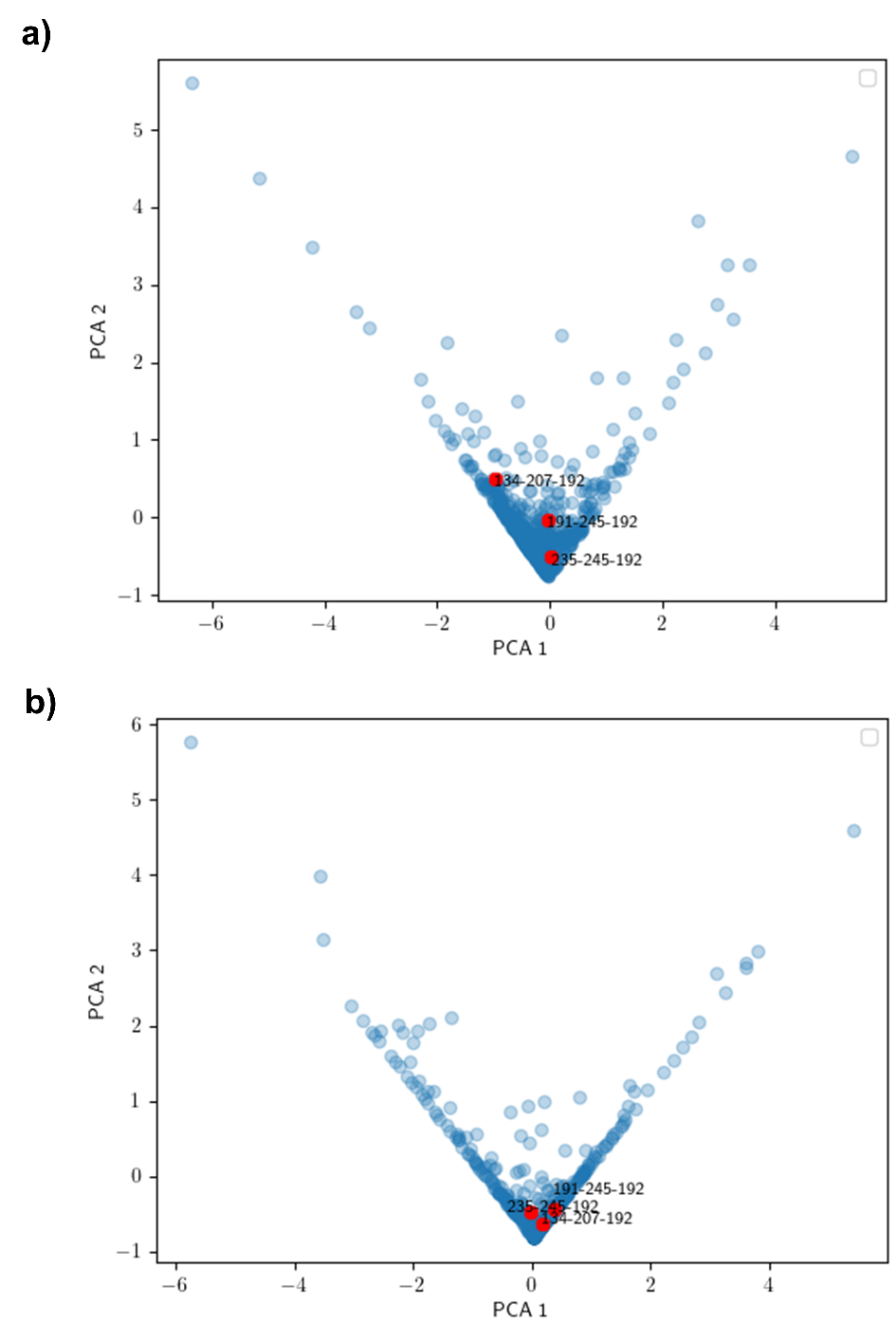
**Figure S5**

**Figure S5.** Similarity analysis for the 30.42 million DEL dataset without INSR indicator enhancement. a) IPCA visualization based on anti-pY1355 data against anti-INSR data. b) IPCA visualization based on anti-pY1361 data against anti-INSR data.

## **Figure S6**

**
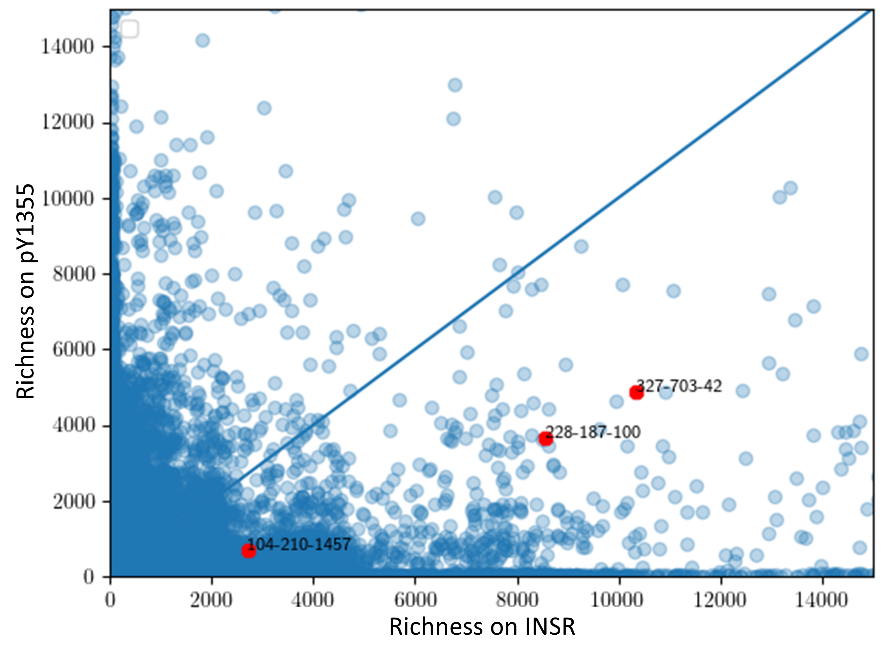
**

**Figure S6** AEC-based DBSCAN filtering for selection of a 1.033 billion DEL against cellular INSR. As deviated data have been removed in BDF, no outliers were identified by DBSCAN and the selection results before and after DBSCAN filtering share the same plot of the richness values as no outliers were removed at this step.

## **
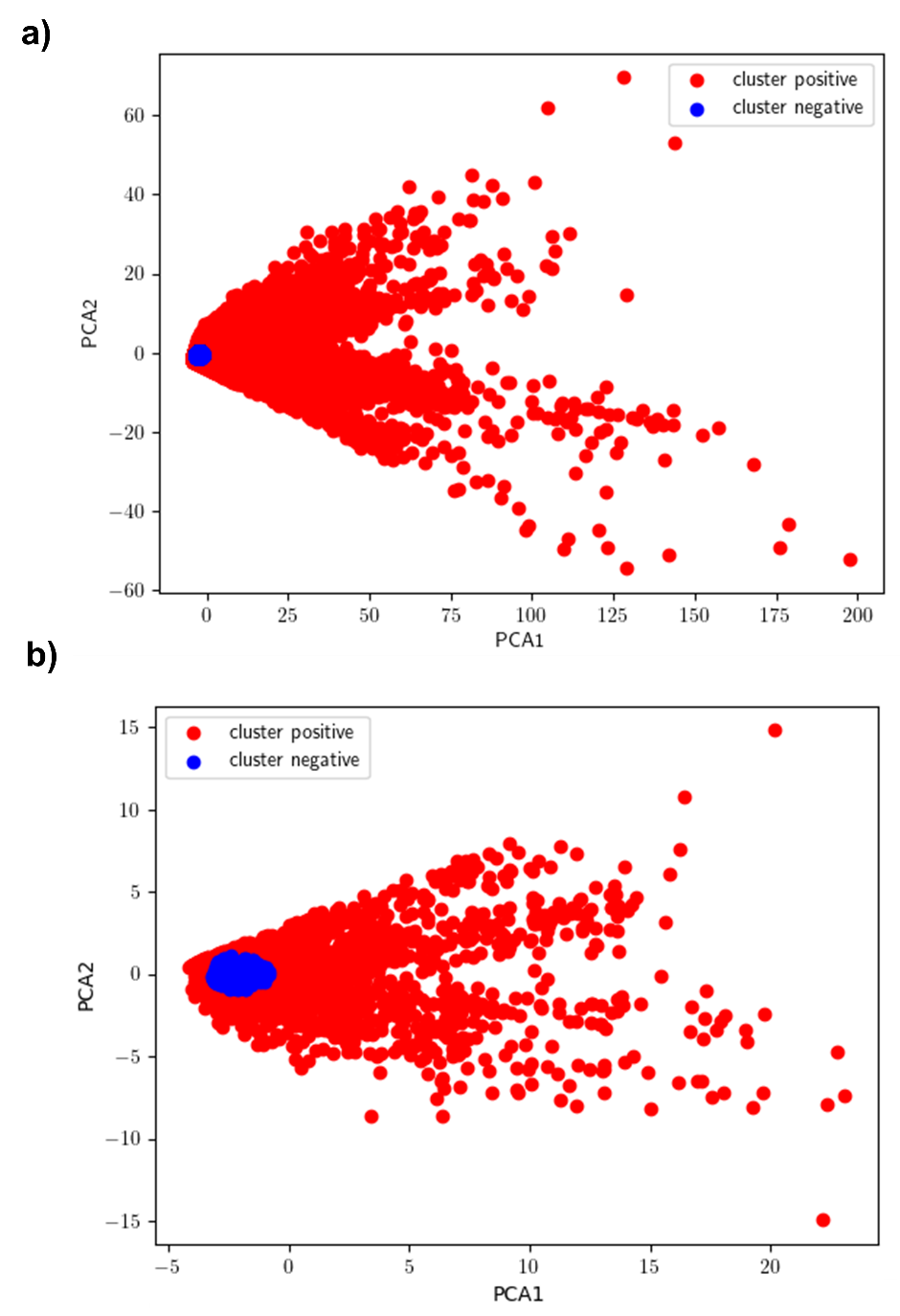
Figure S7**

**Figure S7.** RBF-kernel-based OCSVM classification for selection of the 1.033 billion DEL against cellular INSR in a) level 0 algorithm and b) level 1 algorithm. The negative candidates are marked in blue, while the positive clusters are highlighted in red. The OCSVM results are visualized by IPCA. PCA1/PCA2: descriptive features regarding compound characteristics are IPCA-reduced to 2 dimensions.

##
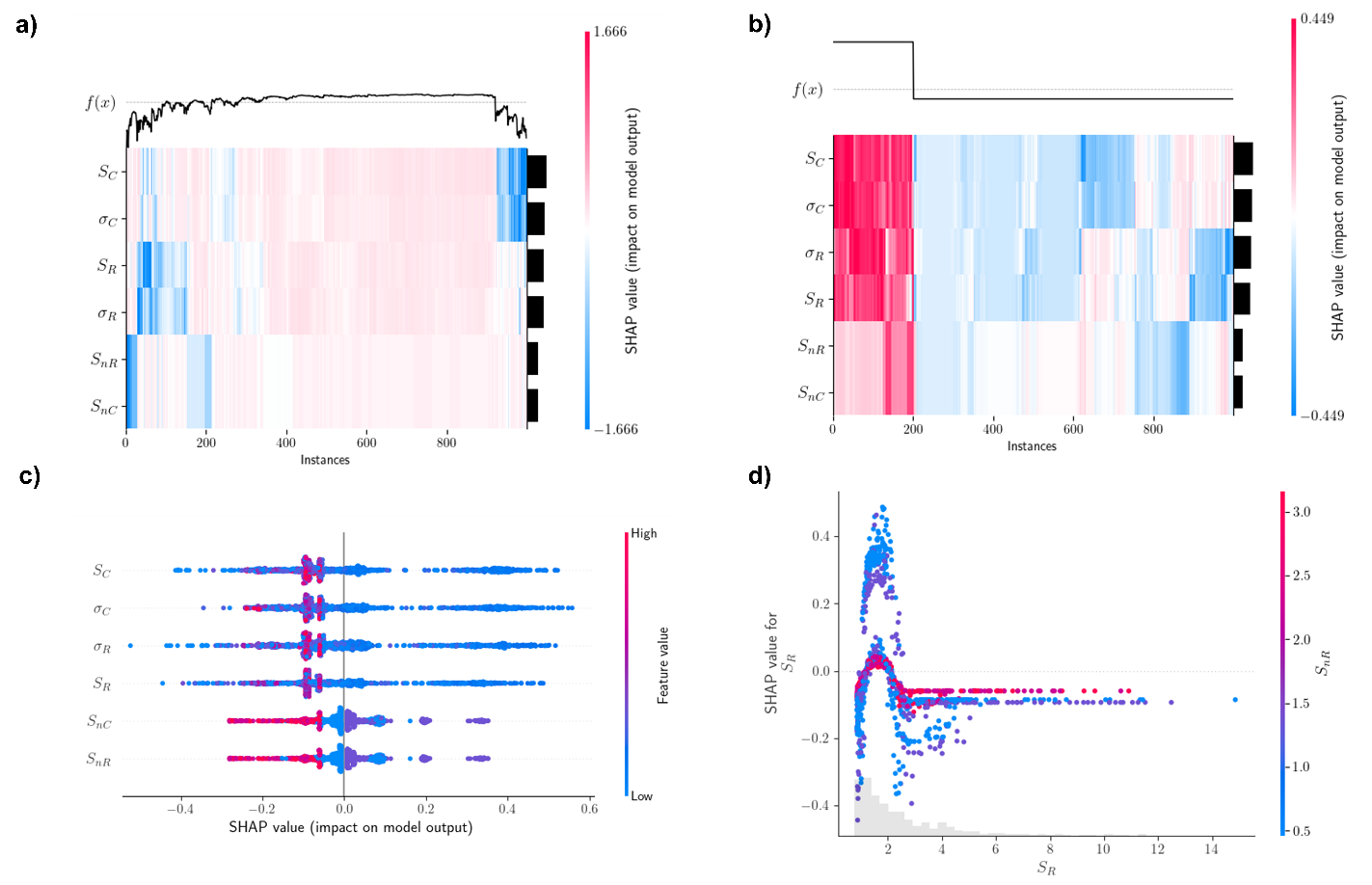
**Figure S8**

**Figure S8.** Shapley value analysis for the 1.033 billion DEL dataset in level 1 algorithm. a) Heatmap analysis ordering by absolute mean of SHAP values. b) Heatmap analysis ordering by sum of SHAP values. c) Beeswarm analysis ordering by sum of SHAP values. d) Dependencies of SHAP values of $S_{R}$.

## **
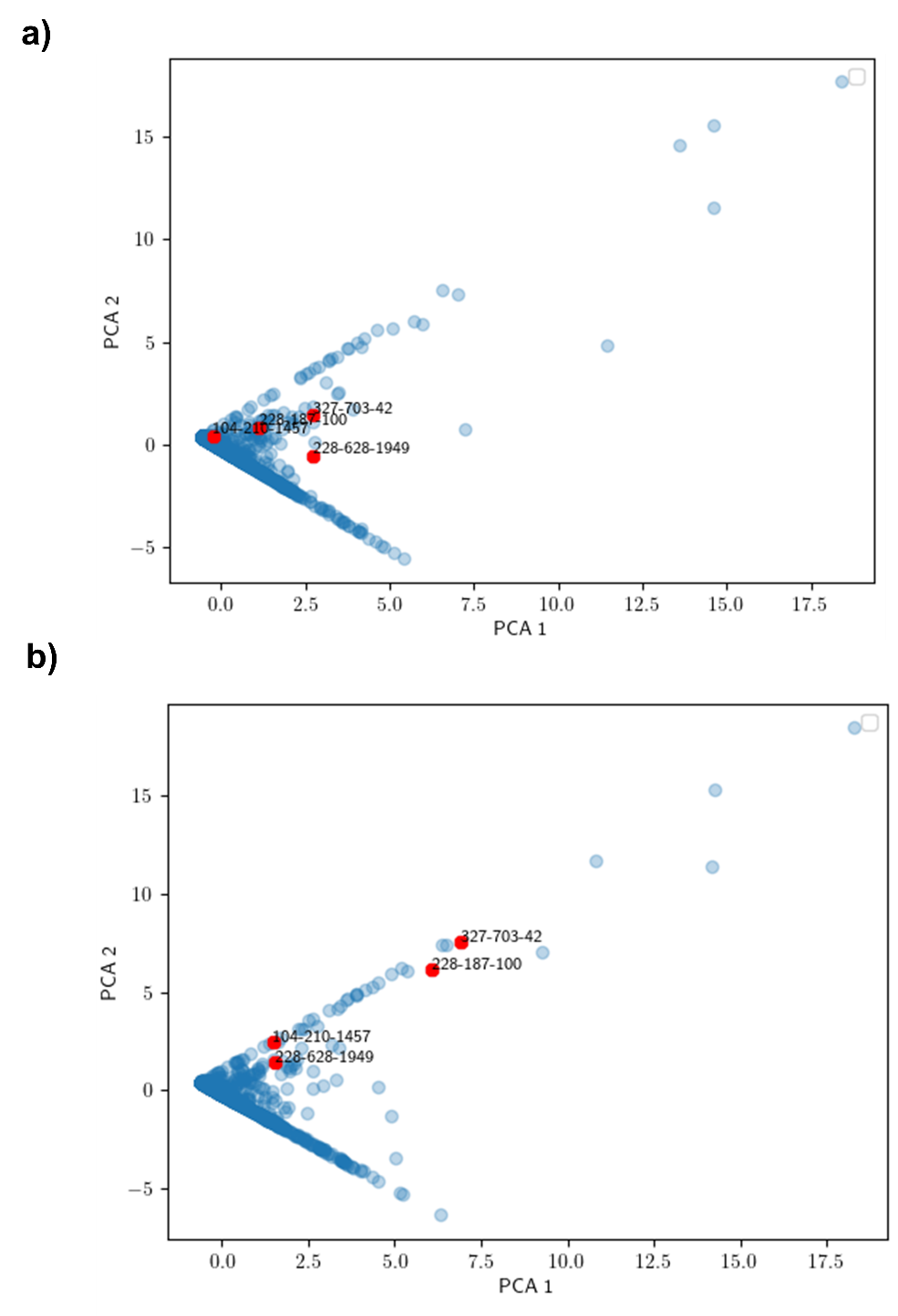
Figure S9**

**Figure S9.** IPCA clustering for the 1.033 billion DEL dataset before similarity analysis. a) IPCA clustering based on anti-pY1355 data against anti-INSR data. f) IPCA clustering based on anti-pY1361 data against anti-INSR data. x-axis recapitulates compound features in anti-INSR selection; y-axis recapitulates compound features in anti-phosphorylated INSR selection. Example compounds were highlighted in red.

## **
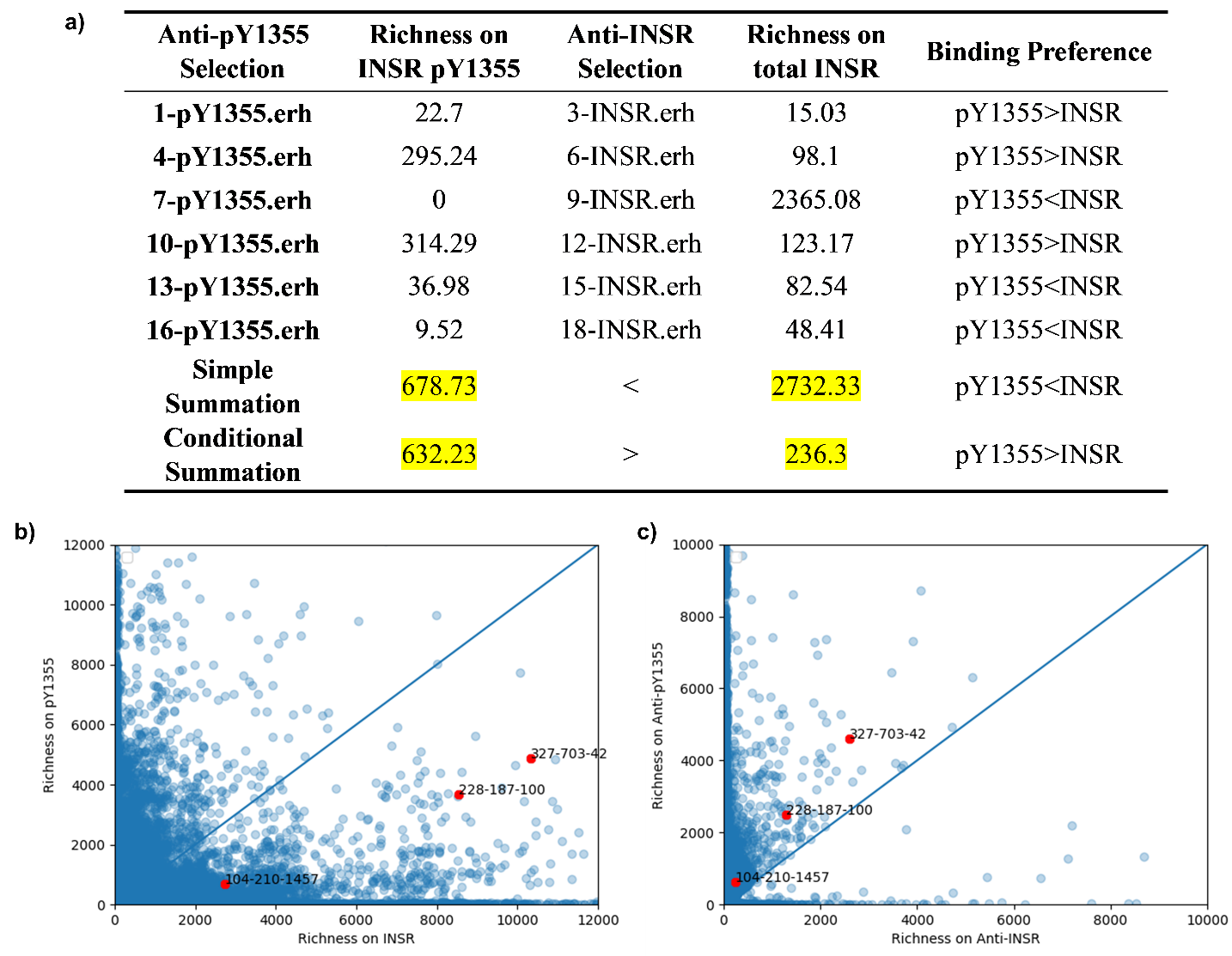
Figure S10**

**Figure S10.** Comparison of simple summation with conditional summation with the 1.033 billion DEL against cellular INSR. a) The performance of a presented example 104-210-1457 in simple summation and conditional summation. b) Plot of the richness values of the selection results with simple summation. c) Plot of the richness values of the selection results with conditional summation. The direct comparison of total richness indicated that the compound performed weaker on pY1355 than on INSR. This discrepancy mainly resulted from a single pY1355 file (7-pY1355.erh) with zero richness, whereas a high richness value of 2365.08 was given in the corresponding INSR file (9-INSR.erh). In contrast, the pY1355 richness outperformed INSR data in 3 out of 6 replicates (≥ 50%), thus those pY1355-superior replicates (1-pY1355.erh, 4-pY1355.erh, and 10-pY1355.erh) contributed a total anti-pY1355 richness of 632.23 and a total anti-INSR richness 236.3 in the conditional summation, demonstrating that the compound was indeed more favorable by INSR pY1355.

##
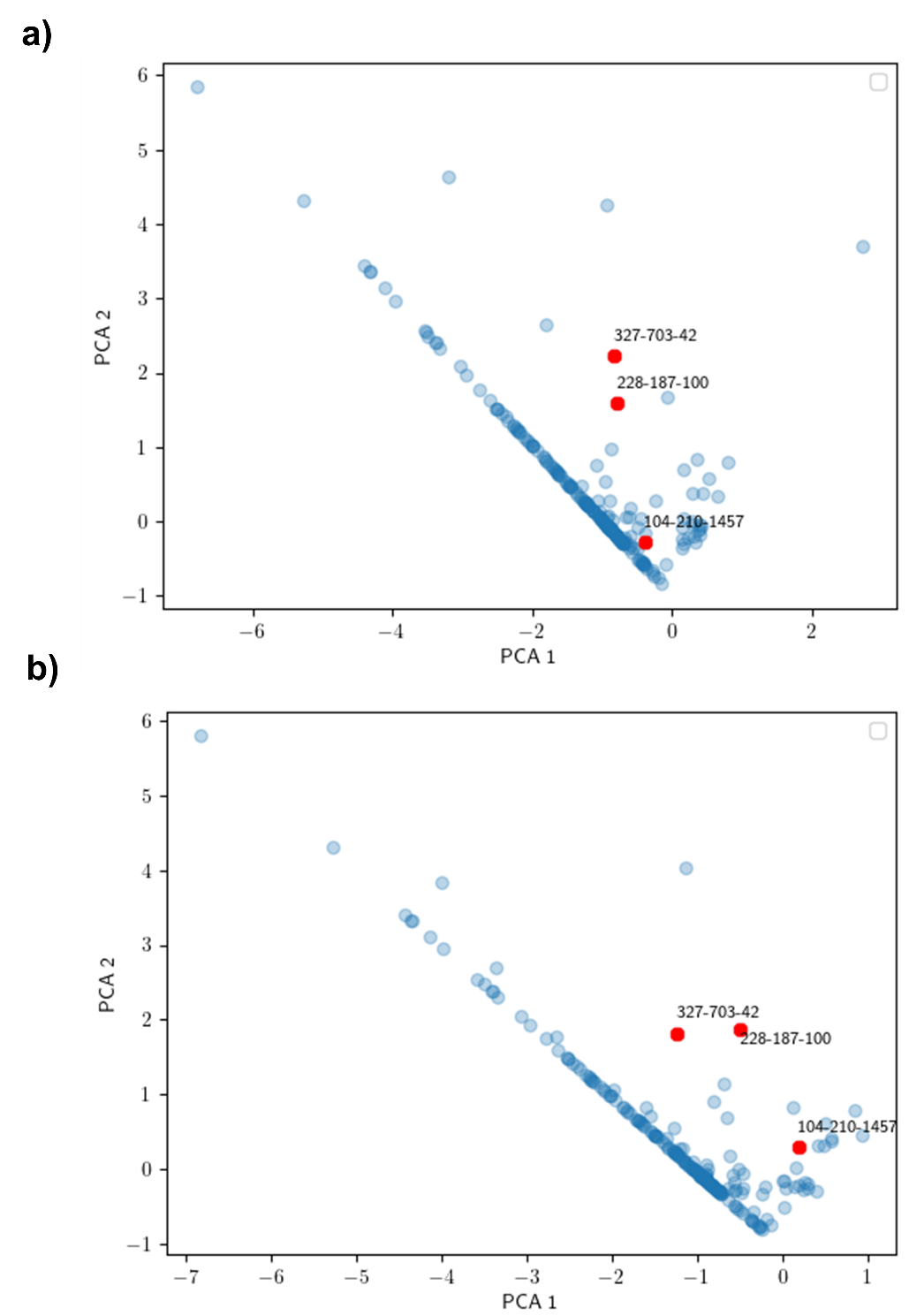
**Figure S11**

**Figure S11.** Similarity analysis for the 1.033 billion DEL dataset without INSR indicator enhancement. a) IPCA visualization based on anti-pY1355 data against anti-INSR data. b) IPCA visualization based on anti-pY1361 data against anti-INSR data. The compounds L1, L2 were positioned at left region to the hyperplane in anti-pY1355/1361 and L3 in anti-pY1355.

## **Figure S12**


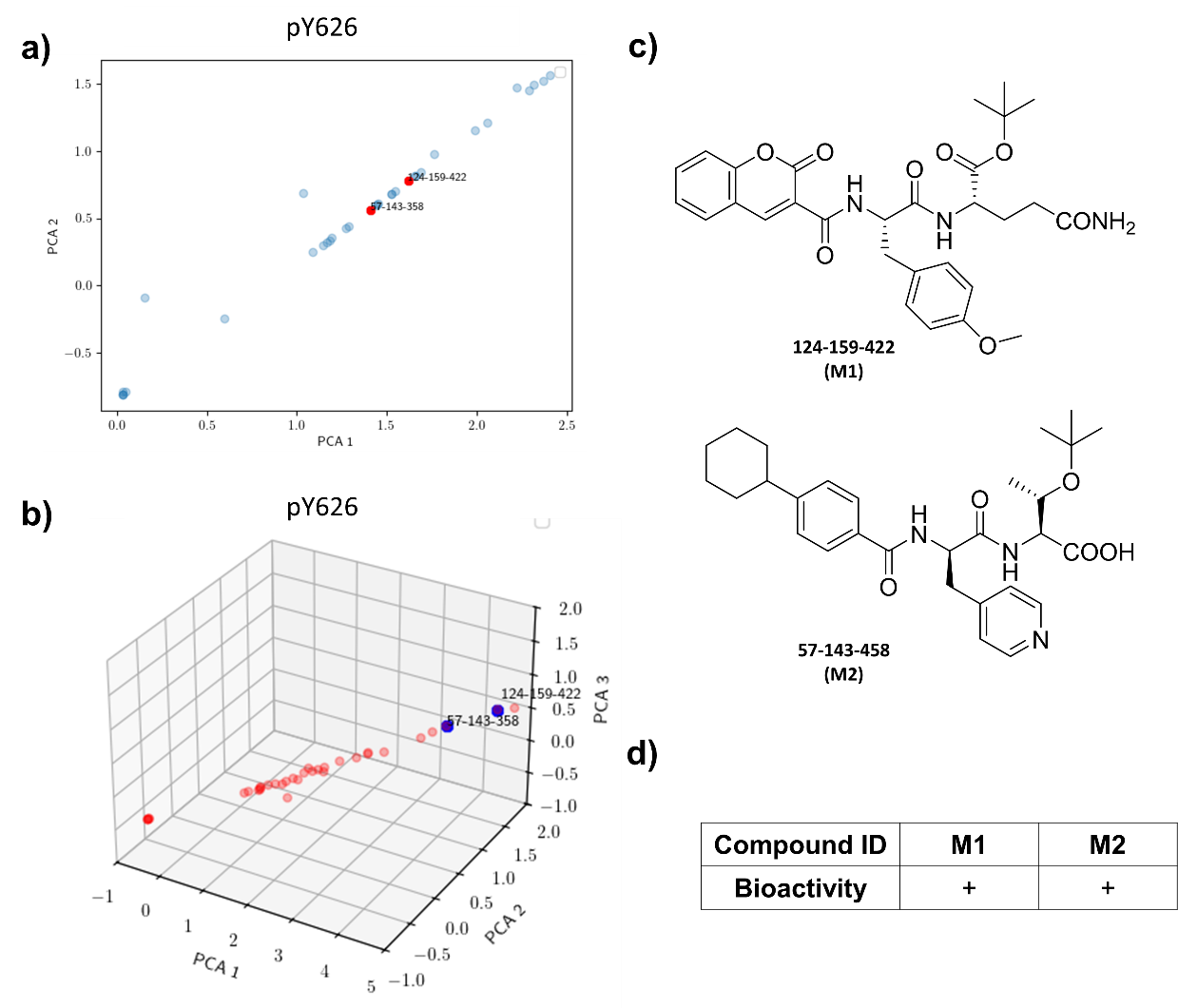


**Figure S12.** Similarity analysis accurately predicted hit compounds from the 30.42 million DEL selections against TPOR on live cells. a) 2-D IPCA visualization based on anti-pY626 data against anti-TPOR data after similarity analysis with TPOR indicator enhancement. b) 3-D IPCA visualization based on anti-pY626 data against anti-TPOR data after similarity analysis with TPOR indicator enhancement. c) Structures of the hit compounds. d) Bioactivity readouts after experimental hit validation. +: bioactive; -: no bioactivity.

# **Methods**

**Raw file decoding and pre-processing.**

All raw data (fastq files) were transformed into processed datasets of clean reads by using a custom method reported by Neri *et al*.^1^ For each experiment, a collection of sequencing counts and code information for all library members were obtained for subsequent data processing. The compound richness $R_{i}$ was calculated from abundance counts $C_{i}$ as below:

$$R_{i}=\frac{\frac{C_{k}}{\sum C_{k}}}{\frac{C_{0k}}{\sum C_{0k}}}$$

$C_{k}$ is the abundance count of post-selection DEL compound $k$ in repeat$i$; $C_{0k}$ is the abundance count of pre-selection DEL compound $k$.

All zero valued DEL compounds in the sum of richness, abundance count, and standard deviation were excluded from the dataset before calculating the Layer 3 descriptors. After obtain all involved Layer 2 and Layer 3 Descriptors with the corresponding formula as listed in **Table 1**, the normalization was performed following the standard normalizer.^2^ The normalized descriptor $X_{norm}$ was calculated from the original descriptor as below:

$$X_{norm}= \frac{x- \mu}{\sigma}$$

$x$ is the descriptor to be normalized, $\mu$ is the average of $x$, $\sigma$ is the standard deviation of $x$.

**Block of descriptors filtering.**

To enhance filtering effectiveness and balance, we construct **three categories of binding blocks**: 1) effectiveness indicators, involving sum of richness ($S_{R}$) **and sum of abundance count (**$S_{c}$); 2) balance indicators, involving average richness over STDEV ($K_{R}$) and average abundance count over STDEV ($K_{c}$); 3) $\Phi_{bal}$, the balance indicator **characterizing cross-relationships between richness and abundance counts. These blocks integrate both richness- and abundance count-related metrics to conduct DEL data filtering from multiple perspectives.**

**The balance block incorporates three key metrics:**

- $K_{R}$: Calculated by dividing the average richness by its standard deviation (STDEV).
- $K_{c}$: Calculated by dividing the average abundance count by its standard deviation (STDEV).
- $\Phi_{bal}$: Calculated by dividing $K_{R}$ by $K_{c}$.

The metrics $K_{R}$ and $K_{c}$ quantified the balance between effectiveness (indicated by richness or abundance count) and stability (indicated by low STDEV) of a compound; and $\Phi_{bal}$ evaluates the correlation between richness and abundance count, specifically how abundant STDEV-related richness is relative to STDEV-related abundance count for a specific library compound. A high $\Phi_{bal}$ indicates that STDEV-related abundance count correlates strongly with abundant STDEV-related richness.

At level 0 algorithm, all the five descriptors were visited for each library members and the intersection of the respective **top percentage**s were selected out of the total population. Here the top 10% were set for effectiveness descriptors and 40% for balance indicators as reasonable parameters in extracting high-confidence data. Specially, $K_{R}$ and $K_{c}$ values of the filtered compounds were individually investigated, and molecules with either value lower than 1 were defined as non-detected or non-enriched ones and excluded from further processing steps. At level 1 algorithm, $\sigma_{R}$ and $\sigma_{c}$, the STEDV of richness and abundance count respectively, were applied as the filtering criteria and the compounds located on the top lowest percentage were retained. Here, the filtering of STDEVs is refined by selecting the lowest 75% for $\sigma_{R}$ and $\sigma_{c}$, so that the compounds that could not stably bind to the target were filtered out.

**AEC-based DBSCAN for automatic outlier filtering.**

DBSCAN is an unsupervised clustering algorithm that leverages density-based principles to identify non-overlapping clusters by analyzing the reachability and connectivity of data points. It operates by defining clusters around central points, which are densely populated regions, and extends clusters outward to include neighbouring points within a specified radius (ε). The algorithm optimizes cluster boundaries by determining localized central points and their associated neighbourhood radii, ensuring clusters are well-defined and compact. Points that lie outside any identified cluster are classified as outliers or noise. Due to its ability to detect arbitrary-shaped clusters and filter anomalies without requiring prior knowledge of cluster counts, DBSCAN is widely used for identifying background noise and isolating anomalies in datasets.

In DBSCAN, the clusters are defined by the formula below:

*p*≤*ε*; *q*≥*MinPts* (2)

Here, *p* is the density reachable from any object in the dataset, *ε* is the optimized radius from the central point *p* to boundary points; *q* is the number of points within a radius *ε* from *p*; *MinPts* is the minimum number of points required to define a cluster. *ε* represents the maximum distance from *p* to the boundary of a cluster, and only when *q* reachable from *p* is larger than or equal to the minimum points can a cluster be formed around *p*. Points lying exactly at a distance *ε* from the core are classified as boundary points. The points that do not belong to any cluster are identified as noise or outliers. DBSCAN aims to optimize the localization of non-overlapped clusters by determining their core, boundary, and noise points by maximizing *MinPts* while minimizing *ε*.^3^

AEC method is used to automatically adjust *ε* and *MinPts*, the two critical DBSCAN hyperparameters. AEC leverages the density distribution of each dataset and the average pairwise distances among data points to determine these parameters. By repeatedly applying the optimized AEC-based DBSCAN model to filter outliers according to these features, we enhanced the statistical significance and consistency of feature agreements within the input dataset, thereby improving the robustness of downstream analyses.

**OCSVM model using RBF kernel.**

OCSVM is an unsupervised classification model that identifies a target class from another class by constructing an optimized hyperplane. This hyperplane is able to position most of the targeted class above it while maximizing the distance from the origin. The principle of OCSVM is formulated as the following quadratic program:

$$\min_{\omega\in F,\xi\in\mathbb{R}^{\mathcal{l}},\rho\mathbb{\in R}} \frac{1}{2}\left\| \omega\right\|^{2}+\frac{1}{\nu\mathcal{l}}\sum_{i} \xi_{i}-\rho(4)$$

subject to $\left( \omega\cdot\Phi\left( X_{i} \right) \right)\geq\rho-\xi_{i}, \xi_{i}\geq0.$

Here, $\omega$ is a regularized support vector that keeps small in the optimized decision function. $\Phi$ is a feature map between $X\to F$, determined by the kernel function. $\rho$ is the bias factor, and $\xi_{i}$are non-zero slack variables controlled by the parameter $\nu\in(0,1]$. $\mathcal{l\in}\mathbb{N}$ represents the number of observations. $\nu$ sets the upper bound on the "small" region and the lower bound on the support vectors. As $\nu$ approaches 0, the upper boundary of the "small" region tends to infinity, and low $\nu$ values may lead to overfitting. If $\omega$ and $\rho$ can resolve the problem in Equation (5), the decision function is optimized.^4^

$$f\left( x \right)=sgn\left( \left( \omega\cdot\Phi\left( X \right) \right)-\rho\right) (5)$$

In this study, Layer 1 and Layer 2 descriptors were adopted as feature vectors. Since no linear relationships were found among the 10 descriptors, RBF kernel was applied to implicitly map the data distribution into higher-dimensional feature space representations.^5^

$$k\left( X_{i},X_{j} \right)=exp(-\frac{d\left( X,X^{'} \right)^{2}}{2\sigma^{2}}) (6)$$

where $d\left( X,X^{'} \right)$ is the Euclidean distance between vector $X$ and $X^{'}$:

$$d\left( X,X^{'} \right)=\left\| X-X^{'} \right\|=\sqrt{\sum_{i} {(X_{i}-X_{i}^{'})}^{2}} (7)$$

As shown in *Eq. 6*, the RBF kernel $k$ computes the high-dimensional feature space that $\Phi$ mapped into. In OCSVM, $k$ must be maximized to minimize $\omega$. The outliers in the dataset are identified as the "small" group and excluded from the positive candidates.

**SHAP for Shapley value calculation.**

SHAP quantifies the contribution of each feature in the predictions by deriving local linear models within the feature space vicinity of all possible feature coalitions. The calculation of SHAP is described as follows:

$$\phi_{f}\left( v \right)= \sum_{S\mathcal{\subseteq F\backslash}\{f\}} \frac{\left| S \right|!\left( \left| \mathcal{F} \right|-\left| S \right|-1 \right)!}{\left| \mathcal{F} \right|!}(v\left( S\cup f \right)-v\left( S \right)) (8)$$

$\mathcal{F}$ represents the complete set of features, and $S$ is a subset of $\mathcal{F\backslash}\{f\}$. $v\left( S\cup f \right)$ is the value of the coalition with the feature, and $v\left( S \right)$ is the value without the feature. Thus, SHAP is the sum of the weighted differences in the values of coalitions with and without a feature, with weights calculated as the inverse of the multinomial coefficient ${(\begin{matrix} \left| F \right| \\ 1,\left| S \right|,\left| F \right|-\left| S \right|-1 \end{matrix})}^{-1}$.

Since SHAP enumerates and calculates the contribution of each involved feature across all possible coalitions, the computational complexity increases exponentially with increased features and coalition size. In our experiments, we use the SHAP sampling function to downsample the coalition size to 1,000 without replacement for both experimental datasets.

Unlike linear models, RBF kernel-based OCSVM calculates the exponent of Euclidean distances between the targeted lower vector and upper vector, which leads to a non-linear model. To compute the prediction probabilities, the logistic function of the Euclidean distances from the candidate DEL to the hyperplane are used to convert radial-symmetric similarities into a local linear model.

**IPCA visualization.**

In level 0 algorithm, IPCA is not used for filtering but rather to visualize the OCSVM results by simplifying the classified descriptors. For PCA or IPCA method, the direction was identified where the data varies the most within a multivariate dataset, and the correlated variables involved in this subspace were combined in a linear form^6^ to extract features (so called principle components) without labeled outcomes. Specially, in IPCA, memory requirements are minimized by processing datasets in batches, while the time-varying mean is corrected by incorporating additional vectors to maintain accuracy.

In level 1 algorithm, IPCA is employed both to visualize the classified data and cluster hit candidates. The compounds’ performance on INSR is incorporated as benchmark metrics during visualization. The binding features towards phosphorylated INSR is projected to y-direction and the features on total INSR to x-axis, so the candidates could be clustered after dimensionality reduction and value normalization. When assessing similarity scores, IPCA figures out the most active candidates by focusing on those with a positive coefficient factor between PCA 1 and PCA2. This allows us to visualize the classification results with IPCA clustering and the strengthened directional folds that indicates the performance of compounds on phosphorylated INSR over total INSR, as detailed in **Section: INSR comparison indicator and conditional summation**.

**Similarity analysis.**

The similarity analysis is based on hybrid learning across IPCA and OCSVM that generates reduced dimensionality and scattered visualization. Similarities are computed between candidate individuals and the OCSVM’s hyperplane or IPCA central axis according to Euclidean distances. Candidates with greater similarity to these decision boundaries are assigned higher scores. After classified as "active" by the OCSVM model, the cumulative scores derived from the distances are used to predict and rank bioactivity of the candidates. While the scatter plots offer qualitative insights into data distribution, the numerical scores and rankings provide complementary quantitative metrics that enhance interpretability and objectivity in evaluating bioactivity levels.

**INSR comparison indicator and conditional summation**

The denoised dataset were organized into five experimental subsets based on the biological replicates, each containing three cognate selections: anti-pY1355, anti-pY1361 and anti-INSR. To calculate INSR comparison indicators, we compared the richness and abundance count of library members in pY1355 file and pY1361 file with those in INSR file from each biological replicates, which served as a benchmark. If both richness and abundance count in pY1355 or pY1361 exceeded those of INSR, the corresponding compound is deemed to outperform INSR, and its performance indicator was incremented by 1. This process were iterated until all replicates were visited.

In conditional summation, two additional metrics were introduced as bioactivity-biasing markers for filtering. The metrics were constructed by comparing the richness and abundance count of anti-phosphorylated INSR over that of anti-INSR on a fold-wise basis, named phosphorylation preference-fold (R-fold and C-fold respectively; as p-folds collectively). These metrics was incorporated to quantify compound performance across phosphorylated INSR and total INSR. If a compound gave p-folds larger than 1 (i.e. it performed better in pY1355/pY1361 than in INSR) across most replicates, only the metrics from the replicates with a p-fold>1 were retained. Conversely, if a compound performed weaker in pY1355/pY1361 in most replicates, the richness and abundance count values from the weaker folds were summed.

(1) Decurtins, W.; Wichert, M.; Franzini, R. M.; Buller, F.; Stravs, M. A.; Zhang, Y.; Neri, D.; Scheuermann, J. Automated screening for small organic ligands using DNA-encoded chemical libraries. *Nat Protoc* **2016**, *11* (4), 764-780. DOI: 10.1038/nprot.2016.039 From NLM Medline.

(2) de Souto, M. C. P.; de Araujo, D. S. A.; Costa, I. G.; Soares, R. G. F.; Ludermir, T. B.; Schliep, A. Comparative study on normalization procedures for cluster analysis of gene expression datasets. In 2008 IEEE International Joint Conference on Neural Networks (IEEE World Congress on Computational Intelligence), 2008.

(3) Parimala, M.; Lopez, D.; Senthilkumar, N. A survey on density based clustering algorithms for mining large spatial databases. *International Journal of Advanced Science and Technology* **2011**, *31* (1), 59-66.

(4) Scholkopf, B.; Smola, A. J.; Williamson, R. C.; Bartlett, P. L. New support vector algorithms. *Neural Comput* **2000**, *12* (5), 1207-1245. DOI: 10.1162/089976600300015565 From NLM PubMed-not-MEDLINE.

(5) Vempati, S.; Vedaldi, A.; Zisserman, A.; Jawahar, C. Generalized RBF feature maps for efficient detection. **2010**.

(6) Greenacre, M.; Groenen, P. J.; Hastie, T.; d’Enza, A. I.; Markos, A.; Tuzhilina, E. Principal component analysis. *Nat Rev Method Prime* **2022**, *2* (1), 100.
